# Interplay between reactive oxygen species and ERK activation in cervical cancer cells

**DOI:** 10.3389/fcell.2024.1465729

**Published:** 2024-11-19

**Authors:** Karen Andrea Larrauri-Rodríguez, Bertha Alicia Leon-Chavez, Verónica Vallejo-Ruiz, Lourdes Millán-Perez Peña, Paola Maycotte

**Affiliations:** ^1^ Centro de Investigación Biomédica de Oriente (CIBIOR), Instituto Mexicano del Seguro Social (IMSS), OOAD Puebla, Puebla, Mexico; ^2^ Facultad de Ciencias Químicas, Benemérita Universidad Autónoma de Puebla (BUAP), Ciudad Universitaria, Puebla, Mexico; ^3^ Centro de Química, Instituto de Ciencias, Benemérita Universidad Autónoma de Puebla (BUAP), Ciudad Universitaria, Puebla, Mexico

**Keywords:** cervical cancer, reactive oxygen species, ERK, MAPK, cancer, migration, survival

## Abstract

**Introduction:**

Among the types of cancer affecting women, cervical cancer (CC) is a public health problem with high global incidence and mortality rates. It is currently classified into three main histological types: squamous cell carcinoma (SCC), adenocarcinoma (AC), and adenosquamous (ASC) carcinoma. All of them lack a targeted therapy. The primary risk factor for CC is Human Papilloma Virus (HPV) infection, which is known to increase reactive oxygen species (ROS), contributing to malignant transformation and tumor progression. At basal levels, ROS can function as second messengers in signaling pathways, and elevated concentrations have been linked to their overactivation. One of these, the ERK pathway, is implicated in both cell proliferation and differentiation and is often dysregulated in cancer, promoting malignant transformation. Several studies have proposed antioxidant supplementation or ERK inhibitors as potential therapies.

**Methods:**

In vitro studies were performed using CC cell lines. ROS levels were evaluated by flow cytometry; cellular proliferation, death and migration were evaluated using real-time microscopy; cell viability was evaluated with crystal violet staining, and phosphorylated ERK levels were evaluated by Western Blot. A bioinformatic analysis was done in a cervical cancer database.

**Results:**

We elucidate part of the complex interplay between ROS and ERK pathway in CC pro-tumorigenic characteristics. Through bioinformatic analysis, we found distinct ROS and ERK activation patterns across CC tumor samples from different histological types. However, *in vitro*, ROS regulated migration and viability in CC, with no discernible variance based on histological classification. ERK activation, however, differed according to the histological type with SCC displaying increased ERK activation compared to AC and regulating cellular migration in SCC cells.

**Discussion:**

Our study identifies a potential synergistic interaction between ROS and ERK inhibitors, highlighting the therapeutic promise of combinatorial targeting for CC treatment. These findings underscore the importance of personalized approaches aimed at improving the outcomes of CC patients.

## 1 Introduction

Globally, cervical cancer (CC) ranked fourth in both incidence and mortality among cancers affecting women (Bray et al., 2024), and according to the Global Cancer Observatory in Latin America and the Caribbean, it ranked as the fourth leading cause of cancer-related death and the third most diagnosed cancer (Cancer Today, 2024). Unfortunately, CC death rate is 18 times higher in low-income and middle-income countries compared with wealthier countries (Small et al., 2017), being the second leading cause of cancer-related death and the second most commonly diagnosed in women in these countries (Bray et al., 2024). CC is attributed to diverse risk factors that include smoking, high number of childbirths, and long-term use of oral contraceptives (Sung et al., 2021), but its main etiological agent is the persistent infection by HPV, which has been associated with approximately 95% of malignant cervical lesions (Small et al., 2017). HPVs infecting the genital area are classified as low or high-risk based on their oncogenic potential. The 16 and 18 genotypes are high risk HPVs associated with CC development and the most prevalent worldwide (Senba and Mori, 2012). Most HPV infection cases are transient and reach viral clearance in 90% of the cases (Toro-Montoya and Tapia-Vela, 2021), but persistent infection and integration of HPV into the host genome defines precancerous lesions and CC development (Senba and Mori, 2012; Small et al., 2017). CC diagnosis currently relies on histopathological analysis of cervical biopsy. Additionally, staging according to the International Federation of Gynecology and Obstetrics (FIGO) system plays a critical role in determining patient outcomes (Cohen et al., 2019; Yan et al., 2019). The FIGO system classifies CC tumors in stage I to IV, with I referring to local disease without extension to the uterine corpus, and stage IV, representing advanced disease extending to pelvic or distant organs (Isla Ortiz et al., 2023; Bhatla et al., 2021).

CC is histologically classified into three main types: SCC, AC, and adenosquamous (ASC) according to the epithelium where the tumor originates (Small et al., 2017), being AC the CC type with the worst prognosis (Yamauchi et al., 2014). Also, less frequent and rare types such as neuroendocrine or small cell carcinoma can be found (Vora and Gupta, 2018). An integrated genomic study showed that the molecular profile between SCC and AC differs. Mutations in *MAPK1/ERK, EGFR, NFE2L2, HLA-B* have been described in SCC, whereas AC tumors have mutations in *ELF3, CBFB,* and *HER2* (Cancer Genome Atlas Research Network et al., 2017). Other studies have reported mutations in the *KRAS* (Ojesina et al., 2014; Hodgson et al., 2017) and RB1 (Hodgson et al., 2017) genes, specifically in AC tumor samples. Besides, a sub-classification for SCC has been proposed, dividing tumors in keratinizing SCC (kerat SCC) and non-keratinizing SCC (non-kerat SCC) (Cancer Genome Atlas Research Network et al., 2017). In some studies, kerat SCC has been associated with a worse prognosis than non-kerat SCC (Kumar et al., 2009), suggesting differences in tumor characteristics, although this prognostic value has been questioned in other studies (Cancer Genome Atlas Research Network et al., 2017). A subclassification for AC has also been described, dividing it in mucinous, endometrioid, and endocervical AC (Jhingran et al., 2003). Despite novel therapeutic options, chemotherapy and radiotherapy remain as the main therapeutic options for CC patients. While adoptive immunotherapy, membrane receptor blockade, and antiangiogenic agents have shown promise in the treatment of metastatic CC, only a few are directed to specific molecular targets or histological types (Vora and Gupta, 2018; Mauricio et al., 2021; Watkins et al., 2023). Thus, similar therapies for all CC patients are used in the clinic independent of their histological classification or the molecular characteristics of the tumor (Small et al., 2017), highlighting the need for novel, targeted therapies that could be beneficial to patient outcome and to prevent metastasis development.

Several reports have shown that a consequence of HPV infection is an increase in cellular ROS (Cruz-Gregorio et al., 2018b; 2018a), which affect different cancer-related cellular processes (Cruz-Gregorio and Aranda-Rivera, 2021). ROS are products of cellular oxidative metabolism and play a significant role in carcinogenesis and tumor progression by regulating processes like proliferation, differentiation, and adaptive immunity (Sarmiento-Salinas et al., 2021). ROS balance is maintained by cellular antioxidant systems. However, excessive ROS levels can lead to oxidative stress and cellular damage (Chio and Tuveson, 2017). Dysregulation of redox-sensitive signaling pathways, including PI3K, NFκB, and MAPK/ERK, is also implicated in cancer development and progression. The MAPK cascade is particularly influential to cancer cells due to its involvement in proliferation, migration, and oxidative stress response (Cruz-Gregorio and Aranda-Rivera, 2021). Thus, overactivated signaling by ROS has been associated with several pathologies including cancer (Cruz-Gregorio and Aranda-Rivera, 2021; Sarmiento-Salinas et al., 2021), but its association and the implication in tumorigenic characteristics in CC remains unclear. Since oxidative stress is crucial in CC development, and ROS have been related to ERK activation (Sarmiento-Salinas et al., 2021), we investigated the role of ROS in CC cells, focusing on their involvement in proliferation and migration, and explored their potential interaction with ERK activation.

## 2 Materials and methods

### 2.1 Bioinformatic analysis

Previously published ROS-related gene signatures (Sarmiento-Salinas et al., 2019), consisting of a total of 370 genes, and an ERK activation gene signature (Acosta-Casique et al., 2023), comprising a total of 67 genes, were used. mRNA Expression Z scores for both signatures were obtained from SCC and endocervical AC database through cbioportal.org (TCGA, 2017). Samples were classified based on histological criteria according to the WHO classification (Small et al., 2017), a subclassification of CC by the TCGA research network (Cancer Genome Atlas Research Network et al., 2017), and FIGO staging classifications (Isla Ortiz et al., 2011). Gene expression levels were used to construct heatmaps with unsupervised hierarchical clustering and principal component analysis plots (PCA) using Expander 8.0.

### 2.2 Cell culture

CC C33-A, SiHa and HeLa cell lines as well as HaCaT keratinocytes were cultured as follows: HaCaT, C33-A, SiHa and HeLa cells were cultured in Dulbecco`s Modified Eagle`s Medium (DMEM, DMP15-10 Caisson), 10% fetal bovine serum (FBS), penicillin (100 U/mL), and streptomycin (1 mg/mL); CaSki cells were cultured in RPMI (RPP12-10), 10% FBS, penicillin (100 U/mL), and streptomycin (1 mg/mL). Cells were maintained in a 5% CO_2_ atmosphere at 37°C. For ROS inhibition, N-acetylcysteine (previously neutralized to pH 7.0 with NaOH) (NaC, A7250 Sigma-Aldrich) and EUK-134 (SML0743-10 MG, Sigma-Aldrich) were used, using water as vehicle. The maximum volume used for drug treatment did not exceed 3.0%. For MEK inhibition, PD0325901 (PD, PZ0162, Sigma-Aldrich) was utilized and diluted in DMSO so that the final concentration of DMSO in culture did not exceed 0.1%, which has been shown to have no effect on the parameters evaluated in this study.

### 2.3 ROS determination

We seeded 95,000 cells in 12 well-plates. After 24 h, cells were treated with 15 mM or 30 mM NaC, or 25–50 nM PD. After 24 h of treatment, cells were washed and stained with 10 μM dihydroethidium (DHE, 37,291, Sigma Aldrich) for 30 min at 37°C, protected from light. Cells were then washed, trypsinized and centrifuged at 2,500 rpm. The pellet was resuspended in PBS with 3% FBS, filtered and analyzed in a BD Facs Canto II flow cytometer, using the PerCP-A channel (Ex: 488 nm, Em: 675 nm). Data was analyzed using Flow Jo V 10.0 software. For ROS^high^ and ROS^low^ populations, cells were analyzed as described by Sarmiento et al.; we defined 95% of the cell population as ROS^high^ and 5% as ROS^low^ (Sarmiento-Salinas et al., 2019).

### 2.4 Proliferation, death and migration assay

Proliferation, death and migration were assessed using the Incucyte ZOOM real-time microscopy system (Essen Bioscience). For proliferation assays, 5,000 cells (CaSki and HaCaT) or 4000 cells (C33-A, SiHa, HeLa) were seeded in triplicate in 96 well-plates, and after 24 h, treated with NaC at the indicated concentrations. Images were captured every 4 h, for 48 h. Data was evaluated using the Incucyte software and expressed as % cell confluence. Cell death was assessed after 48 h with 10 μM propidium iodide (PI, Sigma P4170) staining, which permeates dead cells with a compromised plasma membrane. Data is expressed as % PI positive cells/% total confluence. For migration experiments cells were plated at a density of 50,000 (C33-A, HeLa) or 60,000 (SiHa, CaSki) cells per well in triplicates in 96 well-plates. After 24 h, a scratch-wound was made using an Essen wound maker. Cells were washed, treated with NaC or PD at the indicated concentration and imaged every 4 h for 48 h. Wound closure analysis was performed using Incucyte 96-well cell migration/invasion software. This software measures and differentiates the wounded from the non-wounded regions using the initial scratch wound mask, which determinates % wound confluence. Data is expressed as % wound healing confluence.

### 2.5 Western blot

Proteins (25 μg) were separated on a 10% acrylamide-bisacrylamide gel and transferred to a PVDF membrane. Membranes were blocked with 5% skim milk in TBS-Tween buffer for 1 h, washed three times and incubated with the following primary antibodies: pERK (4370, Cell Signalling Technology, 1:1,000), ERK (4695, Cell Signalling Technology, 1:1,000), β-Actin (A5441, Sigma Aldrich, 1:5,000) at 4°C overnight. Following incubation, membranes were washed 3 times with 0.05% TBS-Tween and incubated in with the following secondary antibodies: anti-Rabbit (7,074, Cell Signalling Technology, 1:10,000), or anti-Mouse (A2304, Sigma Aldrich, 1:10,000) for 1 h at room temperature. Immunodetection was performed with HRP-kit (Immobilon TM Western, Millipore WBKLS0500) and quantified on a C-DiGit transfer scanner (LI-COR). Densitometry analysis was performed using ImageJ software, and relative intensity of the bands was obtained and graphed. Complete membranes of the cropped images shown in the figures with their corresponding molecular weight markers are shown on Supplementary Figure S8.

### 2.6 Crystal violet staining

In 96 well-plates 5,000 cells per well were seeded in triplicates and incubated overnight at 37°C. Cells were later treated with EUK, NaC or PD at the indicated concentrations. After 48 h, the media was aspirated, and cells were fixed (10% acetic acid, 10% methanol, 80% deionized water) for 20 min at room temperature. The cells were then washed with PBS and stained with crystal violet solution (0.4% Crystal violet, 20% ethanol) for 20 min at room temperature. Cells were washed to remove excess stain and let to dry overnight. Finally, stained colonies were solubilized in acetic acid (30%) and absorbance at 540 nm was quantified on a Synergy4 (Biotek) spectrophotometer.

### 2.7 Statistical analysis

Statistical analysis was performed using GraphPad Prism v5 software. Graphs display data from three to five independent experiments performed in replicates represented as the mean ± standard deviation (SD). For migration assays, biological replicate data from three independent experiments were included for the statistical analysis, and the graphs represent the mean ± standard deviation (SD) of each independent experiment. Data was analyzed using one-way or two-way ANOVA as indicated in the figure legend, followed by a *post hoc* Tukey-test. *p*-values <0.05 were considered statistically significant, with *p < 0.05, **p < 0.01, ***p < 0.001, and ****p < 0.0001.

## 3 Results

### 3.1 ROS levels in cervical cancer cells do not differ between histological types

**FIGURE 1:**
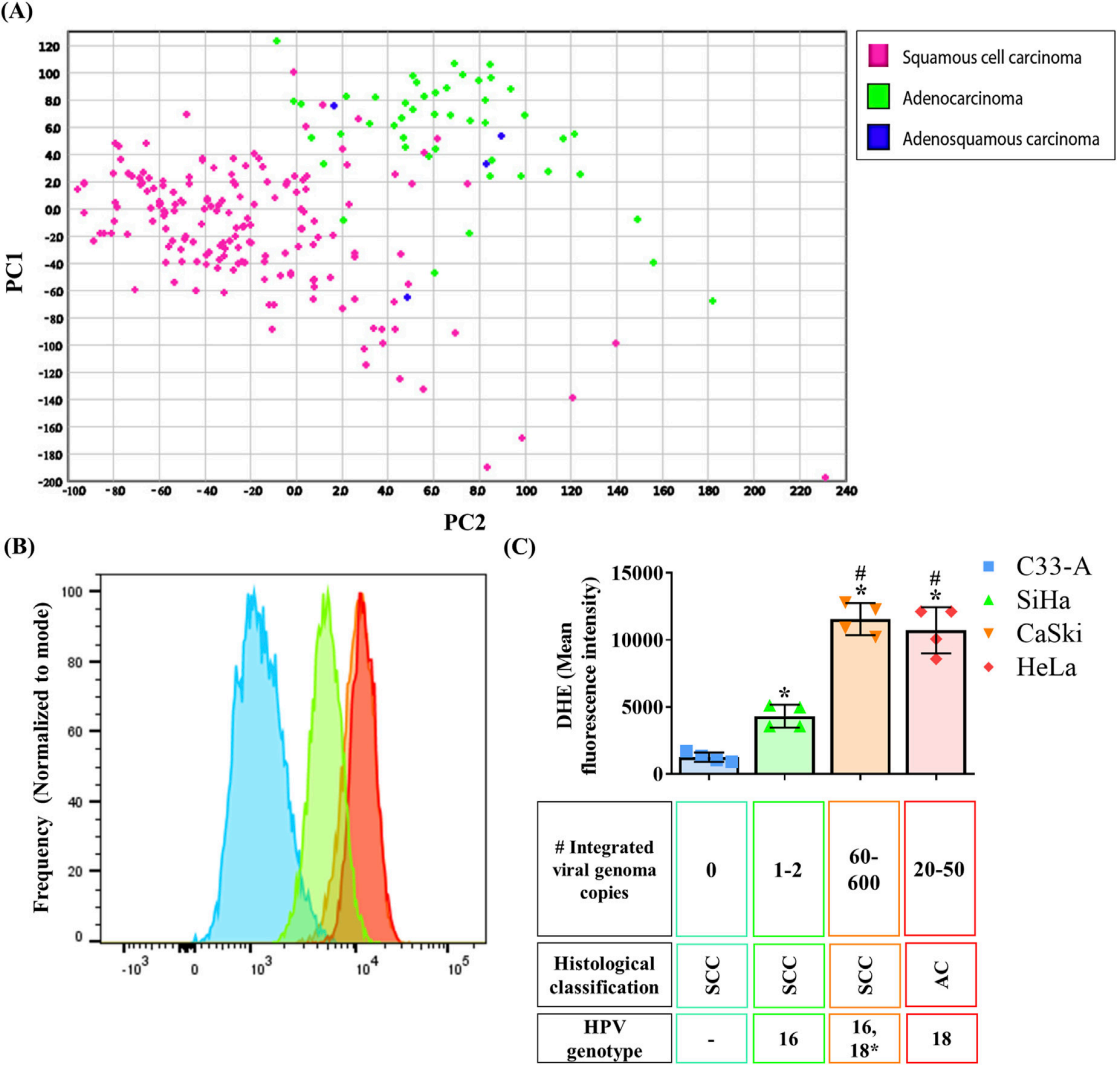
Oxidation on cervical cancer tumor samples and cell lines. **(A)** A ROS production and scavenging related gene signature was analyzed in a TCGA cervical cancer sample database and Principal Component Analysis (PCA) analysis was performed showing two major clusters, one enriched in squamous cervical carcinoma samples (pink), and the second one enriched in cervical adenocarcinoma and adenosquamous samples (green and blue, respectively); PC1 and 2, principal component 1 and 2 respectively. **(B, C)** ROS basal levels were quantified by DHE staining in CC cell lines from different histological classification (SCC, squamous cervical carcinoma; AC, adenocarcinoma), with different integrated viral genome copy numbers, or HPV genotype (16, 18 or 18*, where hybridization with HPV18 sequences has been detected). Graph shows mean ± SD of four independent experiments. One-way ANOVA; Tukey *post hoc*. p < 0.05. * vs. C33-A; # vs. SiHa. HPV, human papilloma virus.

Recent studies have shown that CC patients have elevated oxidative stress markers in serum when compared to healthy controls (Zahra et al., 2021). To assess the oxidant-antioxidant profile in CC, we analyzed a ROS related gene expression signature using a CC tumor sample database. PCA analysis and hierarchical clustering of CC samples classified by histological types are depicted on Figure 1A and Supplementary Figure S1 respectively. We found two major clusters. One cluster was enriched with SCC samples, while the second cluster was enriched with AC and ASC carcinoma. Importantly, some SCC samples clustered with AC rather than with the SCC-enriched cluster. A similar distribution was observed when using CC subclassification, with differences between SCC and AC and no separation between kerat SCC and non-kerat SCC subtypes. This clustering revealed that the SCC samples that clustered with AC were a subgroup of non-kerat SCC (Supplementary Figures S2A). Additionally, we performed the same analysis with samples categorized according to FIGO stage in both SCC (Supplementary Figure S2B) or AC (Supplementary Figure S2C), however, clusters were not observed according to these classifications. Altogether, this analysis suggests that ROS production and management differs between CC histological types and also indicates a small subgroup of non-kerat SCC with similar ROS production and management to AC.

To evaluate potential variations in ROS levels between histological types, we measured ROS levels in CC cell lines from the different histological types, HPV genotype, and number of integrated viral genome copies (Figures 1B, C). For this purpose, ROS levels were measured in each cell line using dihydroethidium (DHE) staining, where increased fluorescence indicated higher ROS levels due to oxidation by species like superoxide anion. C33-A cells exhibited the lowest ROS levels, followed by SiHa, whereas CaSki and HeLa displayed the highest ROS levels (Figures 1B, C). Additionally, we assessed ROS levels in the HaCaT non-tumorigenic cell line derived from human keratinocytes. Surprisingly, HaCaT exhibited the highest ROS levels compared to CC cell lines, similar to CaSki or HeLa cells. (Supplementary Figure S3A). Although no differences were noted in ROS levels across histological types represented by the CC cell lines, our findings suggest a potential association between ROS levels and a greater number of integrated viral genome copies since CaSki and HeLa cells are known to have the highest number of integrated HPV copies (Yee et al., 1985), and displayed the highest ROS levels. This is an observation not previously reported and possibly linked to late HPV infection stages or advanced stages of cancer progression.

### 3.2 Cell proliferation, survival and migration are mediated by ROS in cervical cancer cells

**FIGURE 2:**
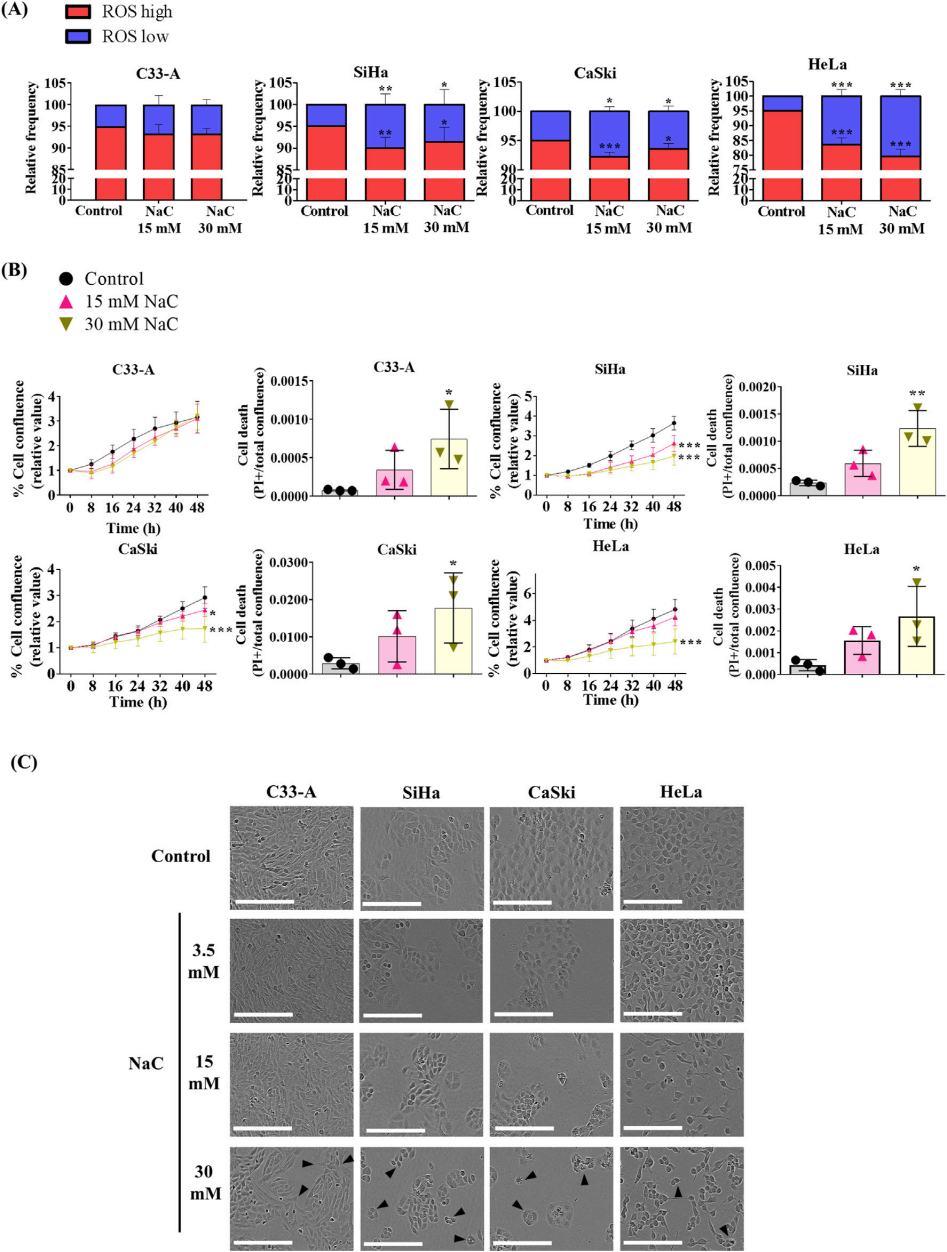
Antioxidant treatment decreased ROS levels, proliferation and survival of HPV positive cervical cancer cells. **(A)** Cervical cancer cell lines were treated with different N-acetylcysteine (NaC) concentrations as shown (15 or 30 mM) and ROS levels were evaluated by flow cytometry after a 24 h treatment. **(B)** Cell proliferation and death were evaluated using a real time imaging System, which show reduced cell proliferation and survival in HPV positive cells after NaC treatment. **(C)** Representative photos following 48 h of NaC treatment, with morphological changes indicated with black arrows, where the white scale bar represents 200 µm. Graph shows mean ± SD of 3-4 independent experiments. Proliferation and death assays were performed in triplicates per experiment. One-way ANOVA **(A, B)** and two-way ANOVA (B, cell proliferation data) were performed; Tukey *post hoc*.; * vs. Control; *p* < 0.05. NaC, N acetylcysteine.

ROS function as second messengers in different cellular processes associated with tumor progression (Cruz-Gregorio et al., 2018b). Given the heightened oxidative stress in CC attributed to HPV infection (Cruz-Gregorio et al., 2018b), we aimed to investigate the impact of ROS on the promotion of cancer features like cellular proliferation (Sarmiento-Salinas et al., 2021). We treated cells with NaC, a glutathione precursor, and ROS levels were assessed with DHE staining after a 24 h NaC treatment. NaC decreased the ROS^high^ and increased the ROS^low^ populations in HPV-positive CC cell lines but not in C33A cells, probably due to its low basal levels of ROS (Figure 2A).

NaC treatment decreased cell proliferation in HPV-positive CC cells and increased cell death in all cell lines tested, as depicted on Figure 2B, Supplementary Figure S4. The images captured following 48 h of treatment revealed a decrease in cell confluence compared to the control (Figure 2C), and morphological changes induced by NaC treatment such as rounded, smaller cells. In parallel with the assessment of high ROS levels in the HaCaT non-tumorigenic cell line, we investigated the impact of NaC on proliferation and survival in these cells. Remarkably, and in agreement with its high ROS levels, HaCaT demonstrated sensitivity to antioxidant treatment, resulting in decreased proliferation and survival (Supplementary Figures S3B, C, S4).We also examined the effect of a second antioxidant, the superoxide dismutase and catalase mimetic (EUK-134) (Shah et al., 2015), on cell survival. Similar to NaC treatment, 25–100 mM EUK-134 decreased cell viability in CC cell lines, as assessed by both real-time imaging and crystal violet staining assay (Supplementary Figure S5).

**FIGURE 3:**
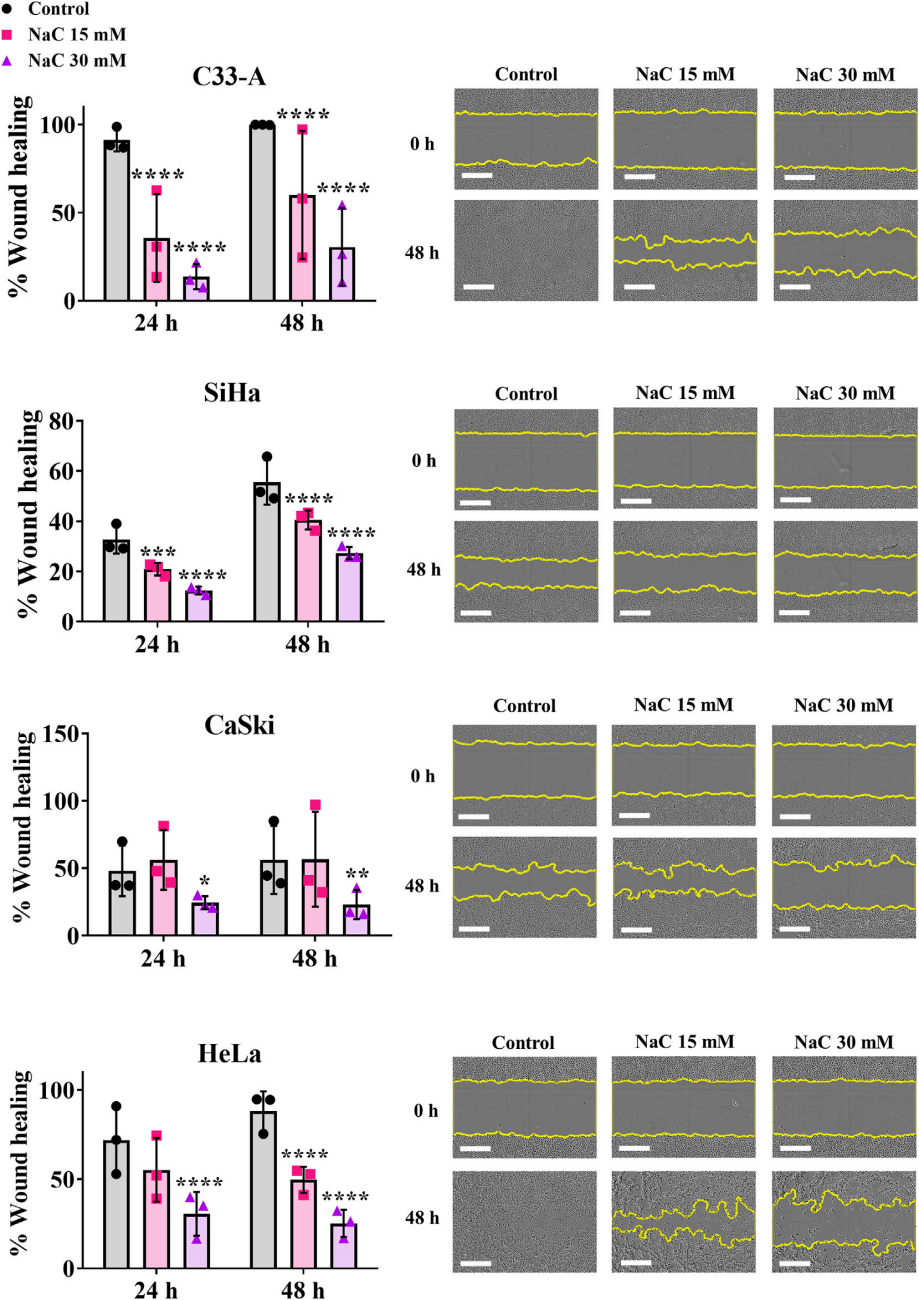
Antioxidant treatment decreased migration of cervical cancer cells. N-acetylcysteine (NaC) effect on migration was evaluated by a wound healing assay, showing the antioxidant treatment decreased migration in all cervical cancer cells following 24 or 48 h treatment. Representative photos taken at 0 or 48 h post treatment are shown. The white scale bar represents 400 µm. Graph shows mean ± SD of three independent experiments performed in triplicates. Two-way ANOVA; Tukey *post hoc*. * vs. Control; *p* < 0.05.

Another process that could be regulated by ROS in cancer is cell migration (Sarmiento-Salinas et al., 2021). NaC treatment led to a significant decrease in cellular migration when measured by a wound healing assay in all CC cell lines (Figure 3). Interestingly, while the treatment with NaC affected migration and cell death in C33-A, there was no significant reduction in ROS levels (Figure 2A). The findings indicate that NaC has a different effect on this specific cell line: it might reduce ROS levels at time points that were not studied, it could lead to small changes in ROS levels that were not detected by DHE staining or the effect of NaC on this cell line is independent of its antioxidant activity. On the other hand, NaC treatment reduced the population of SiHa, CaSki, and HeLa cells with high ROS levels and also affected their proliferation, survival, and migration. This underscores the important role for ROS in controlling these processes in CC cells.

### 3.3 ERK activation differs according to the CC histological type

**FIGURE 4:**
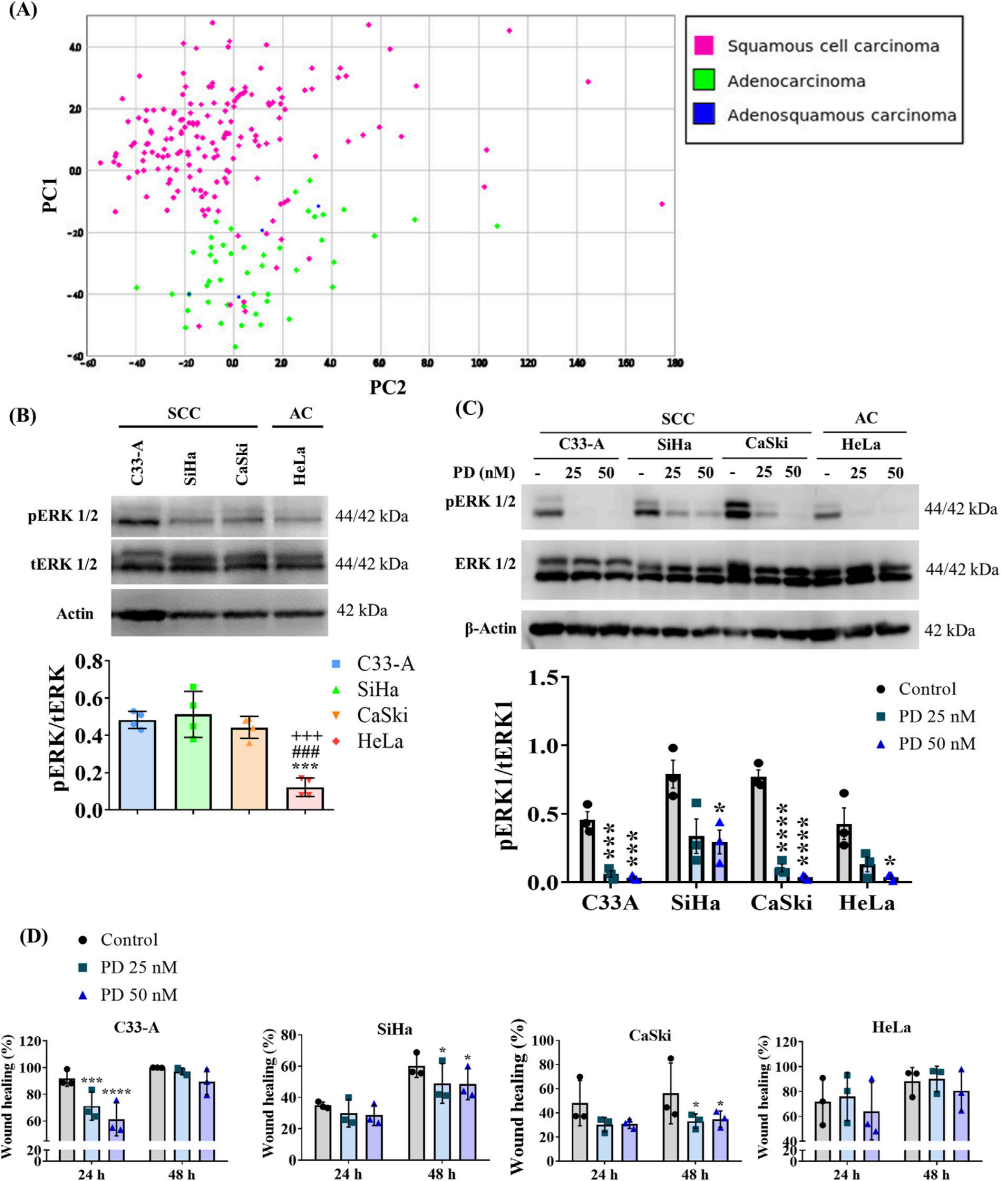
ERK activation differs between cervical cancer histological types and MEKi treatment decreased cell migration in SCC. **(A)** ERK activation gene signature was analyzed in a TCGA cervical cancer sample database, and principal component (PC) analysis was performed revealing two major clusters, one enriched by squamous cervical carcinoma (pink) and a second one enriched in cervical adenocarcinoma and adenoesquamous samples (green and blue, respectively). **(B)** Basal levels of ERK phosphorylation were evaluated by Western blot in cervical cancer cell lines and revealed differences between histological types. **(C)** 1 h treatment with MEK inhibitor PD0325901 (PD) (25 or 50 nM) decreased ERK phosphorylation in cervical cancer cell lines. **(D)** The effect of the MEK inhibitor on migration was evaluated by a wound healing assay, showing that MEKi treatment decreased migration in squamous cell lines. Graph shows mean ± SD of 3-4 independent experiments. One-way ANOVA **(B)** or Two-way ANOVA **(D)**; Tukey *post hoc*. * vs. Control or C33A; # vs. SiHa; + vs. CaSki; *p* < 0.05. SCC, squamous cell carcinoma; AC, adenocarcinoma.

Several signaling pathways are sensitive to redox regulation, like the MAPK cascade (Sarmiento-Salinas et al., 2021), which has been reported as altered in CC (Ojesina et al., 2014; Zou et al., 2017; Cruz-Gregorio and Aranda-Rivera, 2021). To dilucidated the ERK activation profile in CC, we examined an ERK activation gene signature using the same CC tumor sample database mentioned previously. Hierarchical clustering and PCA analysis of samples according to their histological classification are illustrated in Figure 4A and Supplementary Figure S6 respectively. The findings closely mirror those obtained with the ROS-related signature, revealing two major clusters that stratify tumor samples based on histological types (SCC and AC/adenosquamous). Also, a small number of SCC clustered with AC and adenosquamous samples rather than with the other SCC. Additionally, we conducted an analysis of this ERK activation signature with samples categorized according to their histological sub-classification or FIGO stage (Supplementary Figure S7); however, no distinct clusters emerged based on these classifications. This suggests that ERK activation varies among histological types but not among histological subclasses or FIGO stages.

Given that ERK phosphorylation (pERK) serves as an indicator of its activation (Acosta-Casique et al., 2023), we assessed basal levels of pERK through Western blot analysis in CC cell lines. Our results demonstrate differences in ERK activation between CC cell lines from different histological classifications, regardless of HPV genotype or integrated viral genome copy number. C33-A, SiHa, and CaSki, classified as SCC, exhibited the highest pERK levels compared to HeLa, classified as AC (Figure 4B; Supplementary Figure S8A). We further investigated the phosphorylation levels of ERK in the HaCaT cell line, as previously mentioned. We observed elevated pERK levels in HaCaT cells, like those found in SCC cell lines (Supplementary Figures S3D, S8A).

### 3.4 ERK modulates migration in SCC cell lines

ERK activation has been implicated in tumor promotion (Luna et al., 2021; Acosta-Casique et al., 2023); therefore, we investigated whether ERK activation was promoting cellular migration in CC cell lines. We treated CC cells with the specific MEK inhibitor (MEKi) PD0325901. Given that MEK is a specific kinase for ERK (Acosta-Casique et al., 2023), MEKi induced a decrease in ERK phosphorylation in CC cell lines when administered for 1 h at concentrations of 25 or 50 nM (Figure 4C; Supplementary Figure S8B). To evaluate the effects of ERK on cellular migration, we performed a wound healing assay in CC cells treated with PD for 48 h and assessed migration using a real-time imaging system. Our results revealed that the MEKi significantly reduced migration in SCC cell lines but not in the HeLa cell line, suggesting an important role for ERK on the migration of SCC cells (Figure 4D; Supplementary Figure S9). Importantly, decreased migration in the C33-A cell line was only transient (24 h) and cells recovered their migratory ability at 48 h.

### 3.5 Inhibition of ROS and ERK differentially affect CC cell viability

**FIGURE 5:**
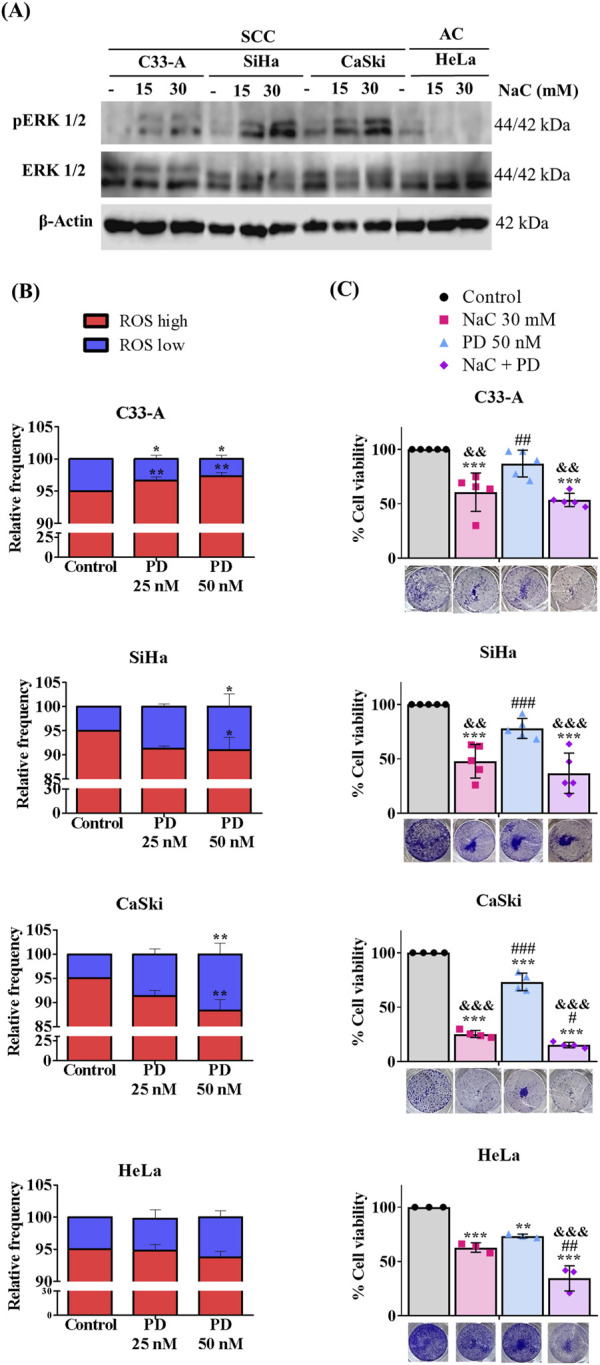
Inhibition of ROS and ERK differentially regulate viability in CC cells. **(A)** Western blot for pERK was performed following N-acetylcysteine (NaC) (15 or 30 mM) treatment for 24 h. **(B)** Cells were treated with MEKi (PD) (25 or 50 nM), for 24 h, and ROS levels were evaluated with DHE staining. **(C)** NaC (30 mM) or/and PD (50 nM) effect on survival was evaluated at the indicated concentrations. Survival was affected by NaC in all cell lines, but MEKi or combined treatment only affected CaSki and HeLa cell survival, suggesting a synergistic effect. Graph shows mean ± SD of 3-5 independent experiments. Survival assays were performed in triplicates per experiment. One-way ANOVA; Tukey *post hoc*. * vs. Control; # vs. NaC; and vs. PD; *p* < 0.05. SCC, squamous cell carcinoma; AC, adenocarcinoma.

Given the elevated levels of ROS in CC cells (Cruz-Gregorio et al., 2018b), and the observed association of ERK activation with the SCC classification (Ojesina et al., 2014; Zou et al., 2017), we decided to evaluate any potential synergistic interaction between ROS and ERK in regulating key cellular processes such as proliferation and survival. We first evaluated whether ROS influenced ERK phosphorylation by treating CC cells with the antioxidant NaC for 24 h and examined pERK levels by Western blot. The results are illustrated on Figure 5A, Supplementary Figures S8C, S10. A tendency to increase pERK levels was observed in SCC, particularly SiHa cells upon NaC treatment, suggesting a negative regulation of basal ROS levels on pERK. AC (HeLa) cells decreased ERK phosphorylation upon NaC treatment, suggesting ERK activation could depend on ROS levels in this particular cell line. Subsequently, we investigated whether ERK regulated ROS levels by treating CC cells with the MEKi and evaluating ROS populations. As illustrated on Figure 5B, our findings indicated a distinct effect of ERK inhibition on ROS populations across the different cell lines. MEKi increased ROS levels in C33-A, decreased ROS levels in SiHa and CaSki and did not affect HeLa cells. These results imply a regulatory role of ERK on ROS level maintenance specifically in SCC, negatively regulating ROS levels in the C33-A cells and contributing to ROS production in SiHa and CaSki. Since ROS and ERK were not always related, we evaluated a possible synergistic effect of ROS and ERK on cell survival. C33-A and SiHa cell lines demonstrated no significant alterations in viability upon treatment with PD or the combined treatment with NaC and were only affected by NaC treatment. CaSki and HeLa showed decreased viability with NaC or PD-only treatments, and a further reduction with the combined treatment, indicative of a synergistic interaction between ROS and ERK in these cell lines (Figure 5C).

## 4 Discussion

Elevated levels of ROS have long been associated with the development of tumors (Sarmiento-Salinas et al., 2021). Studies consistently show that tumor cells from various cancer types, such as breast cancer and gastric cancer, exhibit higher ROS levels compared to their normal tissue counterparts (Chio and Tuveson, 2017). These elevated ROS levels are often attributed to exposure to risk factors such as smoking, radiation, viral agents, and inflammation, all of which are associated with cancer development (Sarmiento-Salinas et al., 2021). Interestingly, differences in ROS levels have been proposed not only between cancerous and normal tissues (Chio and Tuveson, 2017; Acosta-Casique et al., 2023) but also among histological or molecular subtypes within the same cancer type (Sarmiento-Salinas et al., 2019). For instance, recent studies in breast cancer have demonstrated that the triple-negative subtype displays higher ROS levels compared to hormone-positive-subtypes, which could be related with a worse prognosis of the former subtype (Sarmiento-Salinas et al., 2019). Recent investigations in CC patient serum have revealed elevated levels of oxidative stress markers during tumor progression, especially in advanced stages (Shrivastava et al., 2021), suggesting an important role for ROS in this cancer type. Besides, the presence of the HPV which is particular to this cancer type, has also been associated with increased oxidative stress in CC (Cruz-Gregorio et al., 2018b). However, no significant differences in ROS levels have been reported between histological types or histological subclassification of CC. Recent efforts have proposed a detailed classification system based on proteomic and genomic characteristics, categorizing CC samples in kerat SCC, non-kerat SCC, and AC (Cancer Genome Atlas Research Network et al., 2017). While some evidence has associated kerat SCC tumors with lower survival rates, keratinization itself does not hold prognostic value in the clinic (Kumar et al., 2009; Cancer Genome Atlas Research Network et al., 2017).

Our work delved into bioinformatic predictions using a ROS-related gene signature, revealing CC tumor samples separated primarily according to histological classification, with one group enriched by SCC and the other enriched by AC and ASC samples (Figure 1A; Supplementary Figure S1). However, when tumors were classified according to their histological sub-classification, SCC tumor samples did not segregate into kerat SCC and non-kerat SCC groups (Supplementary Figure S2A). This suggests distinct ROS production and scavenging profiles among histological types but not among SCC subtypes, representing a significant finding. Moreover, a small non-kerat SCC group of samples clustered with AC and adenosquamous samples, suggesting a subset of non-kerat SCC with similar ROS-management characteristics to AC. Nonetheless, further studies in patient samples are warranted to confirm differences in ROS levels between histological types. Furthermore, no discernible differences in ROS production and scavenging were observed when analyzing samples categorized by FIGO staging (Supplementary Figures S2B, C). These findings contrast with previous evidence that reported differences in oxidative stress markers and antioxidant activity between early and advanced stages (Shrivastava et al., 2021), but their evaluation was based on serum levels, whereas ours utilized data from tumor samples and encompassed the entire oxidant-antioxidant profile. This disparity may suggest potential differences between the tumor and circulating oxidant-antioxidant states, underscoring the need for further investigations.

To test these findings in an *in-vitro* system, we assessed ROS basal levels in four CC cell lines, finding C33-A with the lowest and CaSki and HeLa with the highest ROS levels (Figures 1B, C). Although no differences were observed across histological types, a potential link between ROS levels and integrated viral genome copies was noted (Figure 1C). This suggests that the increased levels of ROS in CaSki and HeLa cells could result from an imbalance between oxidants and antioxidants due to the expression of HPV proteins and oncoproteins, as reported by Cruz-Gregorio et al. (Cruz-Gregorio et al., 2018b). While the absence of evidence comparing HPV protein expression complicates establishing a direct association, high-risk HPV, E6 and E7 oncoproteins are known to be involved in the transformation process (Senba and Mori, 2012) and E6 alternative splicing products, such as E6*, has been implicated in oxidative stress (Cruz-Gregorio et al., 2018b; 2018a). Williams et al. observed similar findings, with CaSki exhibiting higher ROS levels and increased E6* expression compared to SiHa (Williams et al., 2014). This suggests that the expression of E6* HPV could mediate of ROS levels in CC. Further studies are needed to confirm E6* effects on oxidative stress in CC biopsy samples and its potential association with tumor progression. Moreover, it should be noted that the CaSki cell line was isolated from a metastatic site (DepMap CaSki Summary, 2024), which could also explain its differences with C33-A and SiHa that also correspond to a SCC but were isolated from a primary tumor (DepMap C33-A Summary, 2024; DepMap SiHa Summary, 2024). Also, a recent study revealed that CaSki cells exhibit higher levels of mesenchymal markers compared to C33-A and SiHa, proposing a mesenchymal phenotype for CaSki cells (Zhang et al., 2021). Since ROS have been shown to induce epithelial mesenchymal transition (EMT) (Lee et al., 2019), it is possible that CaSki cells need elevated ROS levels to maintain their EMT and migratory phenotype. Additionally, variations in ROS markers in tissue samples may be influenced by the stage of the disease, given the fluctuating redox cellular environment during the viral HPV cycle (Cruz-Gregorio et al., 2018b). Several studies have reported elevated oxidation markers levels correlating with cancer progression in various tissues such as colorectal (Sheridan et al., 2009), breast (Curtis et al., 2010) and prostate cancers (Shukla et al., 2020), which also happen to exhibit higher levels of oxidation markers compared to non-cancerous tissue. Concerning CC, a recent study in cervical non-cancerous tissue revealed variability in ROS and oxidative stress (OS) markers between healthy subjects, and demonstrated that the level of DNA damage mirrored the level of ROS in cultured primary cells from the same samples (Katerji et al., 2020). This evidence suggests that further analysis of oxidation markers in tissue samples should reflect ROS and DNA damage levels, but also acknowledges the potential for variation among samples, which could be due to genetic or epigenetic environmental influences (Katerji et al., 2020) or to the presence of HPV in the CC samples. Moreover, possible differences in ROS levels between CC tissue and CC cell lines may exist, as cancer cell lines undergo the transformation necessary for immortalization and culture, which comes along with high proliferation rates (Godbey, 2014). Furthermore, transformed cells have been associated with higher ROS levels than normal cells, and the stimulation of progression of the cell cycle by mutations needed for the cell to be immortalized could involve signaling pathways associated with increased ROS production (Schumacker, 2006). Since our data in CC cell lines does not correlate to our findings in CC tumor samples, which suggests differences in ROS management between different histological types, further experiments need to be performed in tumor samples to confirm this idea.

Given the association of ROS with tumor progression (Zahra et al., 2021), particularly in CC linked to HPV infection, antioxidants have emerged as potential therapies to attenuate tumor growth (Cruz-Gregorio et al., 2018b; Glorieux et al., 2024). Our assessment of the antioxidant NaC on CC cell lines revealed effects consistent with this idea. While NaC treatment decreased migration and increased cell death in the C33-A HPV-negative CC cell line (Figures 2B, 3; Supplementary Figure S4; Table 1), it did not significantly reduce ROS levels or proliferation (Figures 2A, B; Table 1), suggesting potential pleiotropic effects of NaC (Chio and Tuveson, 2017) or limitations in our detection methods. However, EUK also decreased C33-A cell viability, suggesting that although with low basal ROS levels, these cells use ROS for proliferation and migration and plausibly suggesting that the decrease in ROS levels in C33-A cells induced by NaC was not substantial enough to be detected by the method used. NaC treatment in HPV-positive cell lines decreased proliferation, survival, and migration (Figures 2B, C; Supplementary Figure S4; Table 1). Furthermore, a second antioxidant treatment (EUK-134) also decreased viability in all CC cell lines (Supplementary Figure S5), highlighting the necessity of increased ROS production in CC cells for sustaining pro-tumorigenic features, which can be counteracted with antioxidant treatment. The antioxidant NaC, a glutathione precursor, has shown efficacy in various cancer cell types (Chio and Tuveson, 2017) including breast cancer, where it reduces proliferation and induces cell death (Sarmiento-Salinas et al., 2019). Additionally, antioxidant supplementation in newly diagnosed patients has been associated with lower mortality and recurrence rates (Chio and Tuveson, 2017). However, conflicting reports exist, with some lung cancer models suggesting that NaC increases tumor growth, while others indicate a protective effect against cancer development in healthy individuals (Chio and Tuveson, 2017; Sarmiento-Salinas et al., 2021; An et al., 2024). Our data suggests a promising role for antioxidant treatment in the suppression of CC cell proliferation and migration and warrants further testing of this method for CC treatment. Nevertheless, the effects of NaC or other antioxidants should be carefully studied when considering treatment for each type of cancer, as their effects could depend on the tissue, stage or cancer subtype.

**TABLE 1:** Summary of ROS and ERK implication in cellular processes in CC cells.

Cell line	Histological classification	HPV genotype	Integrated viral genome copies	Increased ROS	ROS affect proliferation, death, migration and/or viability?	Increased pERK	ERK affects viability?	ERK affects migration?	ERK regulation of ROS	Combined inhibition of ROS and ERK affect viability	Conclusion
C33-A	SCC	-	0	No	Yes	Yes	No	Yes	Yes	No	ERK is elevated and regulates ROS and migration. ROS regulate survival and migration
SiHa	SCC	16	1–2	Yes	Yes	Yes	No	Yes	Yes	No	ROS are increased (vs. C33-A), regulated by ERK. ROS regulate survival and migration. ERK regulates migration
CaSki	SCC	16, 18^a^	60–600	Yes	Yes	Yes	Yes	Yes	Yes	Yes	ERK is elevated and regulates ROS to regulate survival and migration in a synergistic event. Both ROS and ERK regulate survival, and migration
HeLa	AC	18	20–50	Yes	Yes	No	Yes	No	No	Yes	ROS regulate proliferation, survival and migration. ROS and ERK regulate survival synergistically

^a^ Hybridization with HPV18 sequences has been detected (Yee et al., 1985); SCC, squamous cell carcinoma; AC, adenocarcinoma.

We also evaluated the HaCaT non-tumorigenic cell line which is normally used as a non-tumorigenic control for CC cells since these cells are immortalized human epithelial keratinocytes of easy manipulation, genetic stability, and exhibit normal growth pattern when transplanted into animal models (Breitkreutz et al., 1991; Wilson, 2013). HaCaT cells have been used as a non-tumorigenic control in different types of cancer such as melanoma (George et al., 2013), colorectal cancer (Susan et al., 2023), and cervical cancer (Artaza-Irigaray et al., 2019). In our results, HaCaT cells exhibited ROS levels similar to those of CaSki and HeLa, the CC cell lines with the highest ROS levels, and were also susceptible to antioxidant treatment, leading to decreased proliferation and survival (Supplementary Figures S3A, C, S4). This observation contrasts with reports in other non-tumorigenic cell lines for breast cancer, leukemia, or gastric cancer, where ROS levels are typically lower in non-tumorigenic controls compared to cancer cell lines (Sarmiento-Salinas et al., 2019; 2021). The high ROS levels in HaCaT cells may be attributed to its intrinsic characteristics, such as elevated expression of DUOX2 (The Human Protein Atlas, 2024), a member of the NADPH dual oxidases family, which is a major source of cellular ROS (Zhang et al., 2016). Therefore, further investigation is needed to understand the biological implications of high ROS levels in this cell line, particularly in studies involving oxidative markers. In addition, as mentioned previously, Katerji et al. evaluated cervical biopsies from healthy subjects and observed variations in ROS levels across samples, potentially linked to exogenous, genetic, or epigenetic factors. They highlighted the presence of basal oxidative stress in the cervix (Katerji et al., 2020), which may predispose some women to HPV infection progression and CC development, as oxidative stress has been reported to facilitate HPV infection and persistence (Cruz-Gregorio et al., 2018b). Thus, variations in oxidation markers may be found when analyzed in normal cervical biopsy samples.

One of the redox-sensitive signaling cascades is the ERK/MAPK pathway (Cruz-Gregorio and Aranda-Rivera, 2021), which has been associated with tumorigenesis due to its triggering of processes such as proliferation and invasion (Sarmiento-Salinas et al., 2021). ERK overactivation has been reported in several cancer types, including ovarian, colon, breast and lung cancer, making it an important target in cancer (Guo et al., 2020). Particularly in CC, alterations in the ERK pathway have been detected (Kang et al., 2007; Ojesina et al., 2014; Zou et al., 2017), leading to clinical studies evaluating inhibitors of MAPK pathway such as EGFR inhibitors, Raf modulators, and MEK inhibitors to reduce tumor progression (Manzo-Merino et al., 2014; Vora and Gupta, 2018). Our bioinformatic analysis with an ERK activation gene signature mirror those obtained with the ROS-related signature, suggesting ERK activation differs according to the histological classification in CC (Figure 4A; Supplementary Figure S6). Similar results were found when using the histological subclassification of CC, with no differences between kerat SCC and non-kerat SCC and a small subgroup of non-kerat SCC clustering with AC (Supplementary Figure S7A). This data is consistent with previous reports who noted differential mutation patterns among histological types (Kang et al., 2007; Kumar et al., 2009; Ojesina et al., 2014; Zou et al., 2017). However, no differences were observed in ERK activation according to FIGO staging. Since there is currently little evidence reporting differences in ERK activation between histological types or targeted therapies based on this pathway for CC, further evaluations on tissue samples are needed (Supplementary Figures S7B, C). Furthermore, when basal ERK activation levels were evaluated, we found results resembling those from the ERK gene signature, suggesting that ERK activation is a recurrent pro-tumorigenic characteristic in SCC, as previously demonstrated by Ojesina et al. (Figure 4B) (Ojesina et al., 2014). On the other hand, in AC, a higher prevalence of nonsense mutations, possibly activating, in KRAS and PI3K have been reported, compared to SCC (Kang et al., 2007; Ojesina et al., 2014). In normal cervical tissue, recent reports indicate that the levels of pERK are low. However, levels increase as cervical lesions progress from low-grade to high-grade. This increase appears to correlate with the expression levels of the oncoproteins E6 and E7 (Luna et al., 2021). The evidence and results provided in this work suggest an oncogenic role for ERK in CC, which could be associated with HPV infection. Moreover, in CC cells, we found significant differences in ERK activation between SCC and AC, with SCC being the most affected by the inhibition of this pathway, but this does not exclude an important role for ERK oncogenic activity in AC.

We evaluated pERK levels in the HaCaT cell line, and found that similar to the increased ROS levels, it showed high levels of ERK activation compared to CC cells (Supplementary Figures S3D, S8A). This contrasts with previous reports in other non-tumorigenic control cells like MCF10A for breast cancer cell lines (Acosta-Casique et al., 2023). In this regard, we have not found evidence of whether this is due to intrinsic characteristics of the cell line, since the high levels of ERK phosphorylation in this cell line, to our knowledge, have not been reported until now.

MAPKs haven been implicated in cellular processes such as proliferation, senescence and migration (Sarmiento-Salinas et al., 2021), and some reports have shown that MAPK/ERK inhibition decreased tumor growth *in vivo* (Guo et al., 2020). To evaluate the role of ERK in some pro-tumorigenic characteristics in CC cells, we studied the effect of the MEKi, PD, on tumorigenic processes. PD is a selective inhibitor of MEK (Acosta-Casique et al., 2023). This MAPK kinase phosphorylates and activates ERK enabling it to translocate to the nucleus and activate its targets (Guo et al., 2020). ERK phosphorylation decreased due to PD treatment, indicating effective MEK inhibition (Acosta-Casique et al., 2023) (Figure 4C). We investigated the impact of PD treatment on the migration of CC cells and found the most notable effect on SCC cells, which also showed the highest level of pERK. In the AC HeLa cell line, no significant effect on migration was observed (Figure 4D; Supplementary Figure S9; Table 1). This data indicates that elevated basal pERK levels influence cellular migration, potentially depending on the histological type in CC. Furthermore, although CaSki exhibited higher pERK levels than HeLa, PD treatment affected the survival of both cell lines, suggesting that CaSki needs high pERK levels to sustain migration and survival, while HeLa uses low pERK levels to sustain survival, but not migration (Figure 5C). This effect on migration has been seen in other types of cancer such as breast cancer (Acosta-Casique et al., 2023); importantly ERK/MEK pharmacological inhibition has been implicated in the regulation of HPV oncoproteins allowing malignant transformation and progression (Luna et al., 2021), but no evidence on migration or proliferation has been reported previously for CC. Furthermore, another redox-sensitive signaling cascade that can also be activated by MAPKs is the PI3K/Akt/mTOR pathway (Cruz-Gregorio and Aranda-Rivera, 2021). This pathway is associated with crucial processes such as cell growth, proliferation, survival, metabolism, differentiation (Li et al., 2010) and metabolism, which is why sustained mTOR signaling has been linked to tumorigenesis (Mossmann et al., 2018). However, dual effects have been noted between ROS and mTOR. Reports indicate that low ROS levels can activate mTOR, while high ROS levels may inactivate mTOR signaling, a phenomenon that appears to be tissue-dependent (Li et al., 2010; Cruz-Gregorio and Aranda-Rivera, 2021). Little is known about the relationship between mTOR and ROS in cervical cancer, but recent studies suggest that HPV oncoproteins may be involved in the activation of the PI3K/Akt/mTOR pathway, potentially promoting tumorigenesis (Cruz-Gregorio and Aranda-Rivera, 2021). Therefore, redox-sensitive signaling pathways and their role in various types of cancer should be taken into consideration.

MAPK/ERK signaling involves several phosphorylation and dephosphorylation processes in specific residues of Tyr, Ser and Thr, conducted by kinases and phosphatases (Guo et al., 2020). These kinases and phosphatases possess cysteine residues, susceptible to oxidation in their thiol groups (Cruz-Gregorio and Aranda-Rivera, 2021). ROS have been proposed as signaling entities that regulate this cascade through oxidation reactions, modifying protein activity. This leads to aberrant signaling that promotes pro-tumorigenic characteristics (Chio and Tuveson, 2017). One of the major proposals is that ROS inactivate phosphatases such as DUSP3, SHP-2, PTP1B, leading to constant activation of MAPK signaling (McCubrey et al., 2007; Sies and Jones, 2020; Sarmiento-Salinas et al., 2021). In this work, antioxidant treatment induced a tendency to decrease ERK phosphorylation in HeLa cells, while in the 3 cell lines corresponding to SCC, antioxidant treatment induced a trend to increase phosphorylated ERK levels, particularly in the SiHa cell line (Figure 5A; Supplementary Figures S8C, S10), suggesting that ERK could be differentially regulated by ROS, depending on the cell type. The association between ROS and ERK and their regulatory mechanisms in CC remain unclear, but recent studies have reported that the use of a NOX inhibitor can downregulate the ERK pathway in HeLa cells (Cruz-Gregorio and Aranda-Rivera, 2021), similar to our findings. Other studies have shown that a decrease in ROS could downregulate the p38/MAPK pathway. Thus the regulation of the MAPK pathway under oxidative stress conditions needs further study, particularly considering the presence of the HPV oncoproteins which can also alter the redox state and hence, redox-regulated signaling pathways (Muthuramalingam et al., 2020; Cruz-Gregorio and Aranda-Rivera, 2021; Letafati et al., 2024). Interestingly, despite high or low pERK levels in CC cells, NaC treatment affected ERK phosphorylation, indicating an important role for ROS in ERK regulation. Several studies have proposed ROS as critical regulators of MAPK/ERK signaling in pathologies such as cardiomyopathies (Ko et al., 2015) or some types of cancer (Sarmiento-Salinas et al., 2021), since antioxidants could decrease ERK phosphorylation, but just a few reports have shown the opposite effect (Acosta-Casique et al., 2023). So, the mechanism by which NaC treatment increased ERK phosphorylation remains to be studied. We assessed if ROS levels were affected by ERK inhibition, which revealed ERK negatively regulates ROS levels in C33-A, while it positive regulates ROS in SiHa and CaSki and did not affect ROS in HeLa cells (Figure 5B; Table 1), implying a cell-specific regulatory role for ERK on ROS levels. Finally, when a possible synergistic effect of ROS and ERK on cell survival was assessed, we observed CaSki and HeLa cell survival was diminished when treated with NaC and PD alone, and a further reduction was observed with the combined treatment (Figure 5C; Table 1), which suggests synergistic interaction between ROS and ERK and possibly independent roles for each pathway in these cell lines.

In summary, our findings indicate that ROS play a crucial role in regulating cell proliferation, survival and migration in CC cells despite basal ROS levels, since antioxidant treatment decreased these processes in all cell lines and strongly supporting a therapeutic use for antioxidant treatment for CC treatment. Furthermore, the presence of HPV and particularly the expression of its oncoproteins, seems to be crucial for the induction of ROS in this type of cancer (Figures 1–3). Regarding the interplay between ROS and ERK activation, ROS inhibited ERK activation in SCC cells with high pERK levels, while in AC cells with low pERK, ROS contributed to ERK activation. This suggests a delicate balance in pERK levels in CC cells (Figure 5). On the other hand, ERK activation seems to be involved in the migration of SCC cells, while its role in survival in SCC was only noted in CaSki cells (Figures 4, 5; Supplementary Figure S9; Table 1). Furthermore, a possible synergistic effect was observed on cell survival in 2 cell lines, CaSki and HeLa, the ones with the highest ROS levels and affected by ERK inhibition alone, suggesting tumors with high ROS levels could benefit from ERK and ROS inhibition combined therapy. Thus, the ERK and ROS interaction seems to be context and possibly cell-type dependent and needs further clarification to understand which types of CC could be treated with a combination therapy. Several reports have studied the inhibition of the MAPK pathway for the treatment of cancer (Luna et al., 2021; Acosta-Casique et al., 2023). Besides, other studies suggest that signaling pathways related to redox control in HPV-related cancer are promising targets and have reported the use of different antioxidants as a therapy for CC (Silva et al., 2018), thus combined therapy comprising antioxidants and other molecular targets could be an interesting approach for CC treatment, as long as we elucidate the patients who can benefit from this combined therapy.

Our findings underscore the heterogeneity within CC histological types, particularly highlighting distinct patterns of ERK activation between SCC and AC. While SCC cells exhibit heightened basal levels of pERK, indicative of a pro-tumorigenic phenotype, AC cells displayed sensitivity to ROS and/or ERK inhibition. Furthermore, our study reveals potential synergistic interactions between ROS and ERK signaling pathways, suggesting combinatorial targeting of these pathways as a promising therapeutic strategy. Although we include gene expression data from patient samples in our bioinformatic analysis, our study is limited to *in vitro* results. Thus, the complexity of these interactions and their association with HPV oncoproteins needs further investigation, particularly regarding their impact on invasion and metastasis as well as validation in CC tissue. This will be addressed in future studies. Overall, our work contributes to a deeper understanding of the molecular mechanisms driving CC progression and highlights the therapeutic potential of targeting ROS and ERK signaling for personalized treatment strategies. Future studies exploring these interactions in preclinical models and clinical cohorts will be crucial for translating these findings into clinical applications and improving outcomes for CC patients.

## Data Availability

The original contributions presented in the study are included in the article/Supplementary Material, further inquiries can be directed to the corresponding author.
