# Supplementary material for: Interplay between reactive oxygen species and ERK activation in cervical cancer cells
Source: Front Cell Dev Biol. 2024 Nov 19;12:1465729. doi: 10.3389/fcell.2024.1465729 (PMC11611811; doi:10.3389/fcell.2024.1465729)
Supplement: Supplementary file 1 [file DataSheet1.pdf]

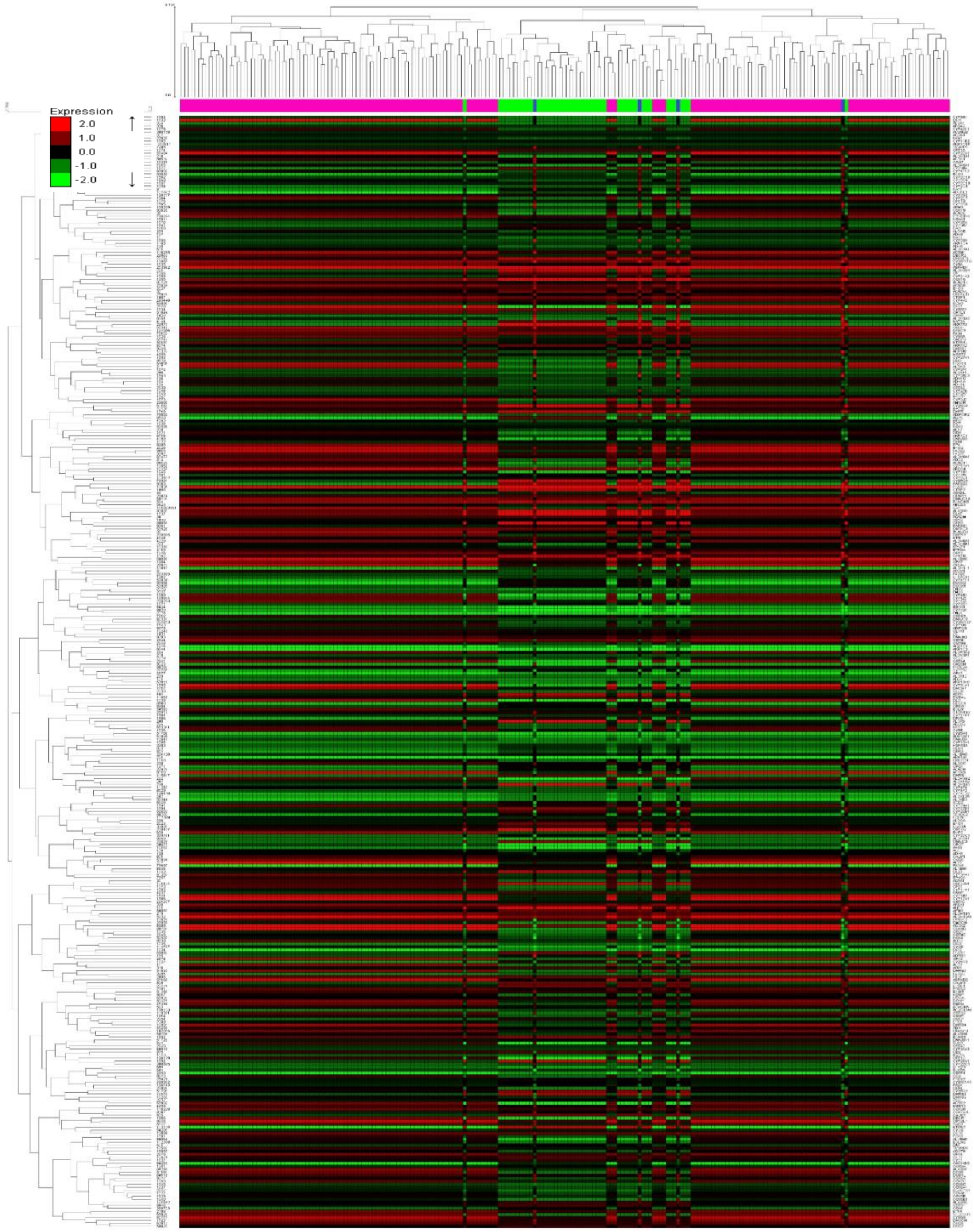

Figure S1. Unsupervised hierarchical clustering displays differences between histological CC types according to a ROS-related gene signature. A ROS production and scavenging related gene signature was analyzed in a TCGA CC sample database, and an unsupervised hierarchical clustering analysis was performed, showing two major clusters of samples enriched by histological classification. Squamous cervical carcinoma (pink); cervical adenocarcinoma and adenoesquamous samples (green and blue, respectively).

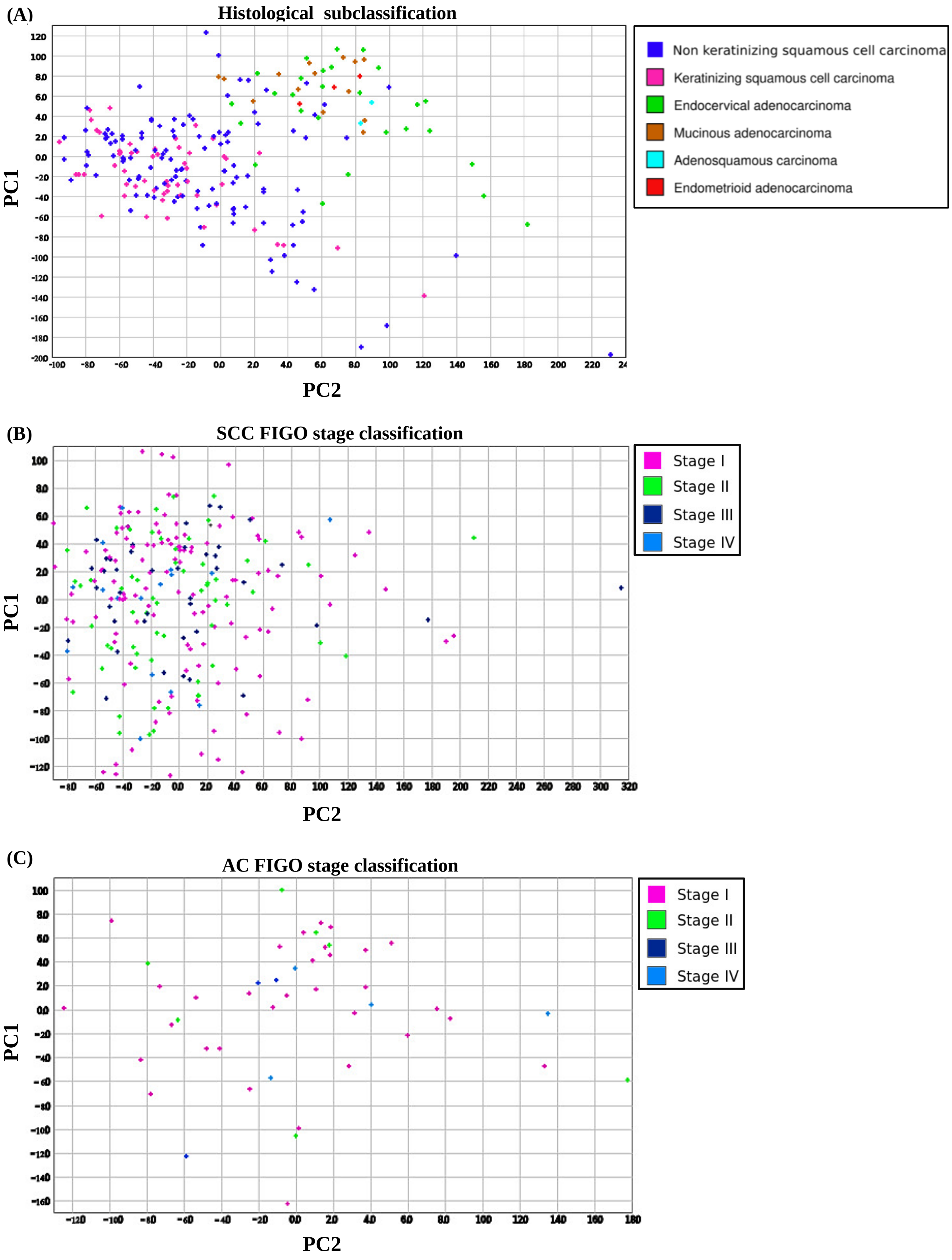

Figure S2. A ROS production and scavenging related gene signature does not separate CC samples according to histological subclassification or stage of the disease (A) The ROS related gene signature was analyzed in CC samples categorized by histological subclassification, but no groups were observed according to this classification. (B, C) Samples were also separated by histological subtype and classified by FIGO stage, the ROS related signature was analyzed and PCA was performed, but no clusters were observed. SCC, squamous cell carcinoma; AC, adenocarcinoma.

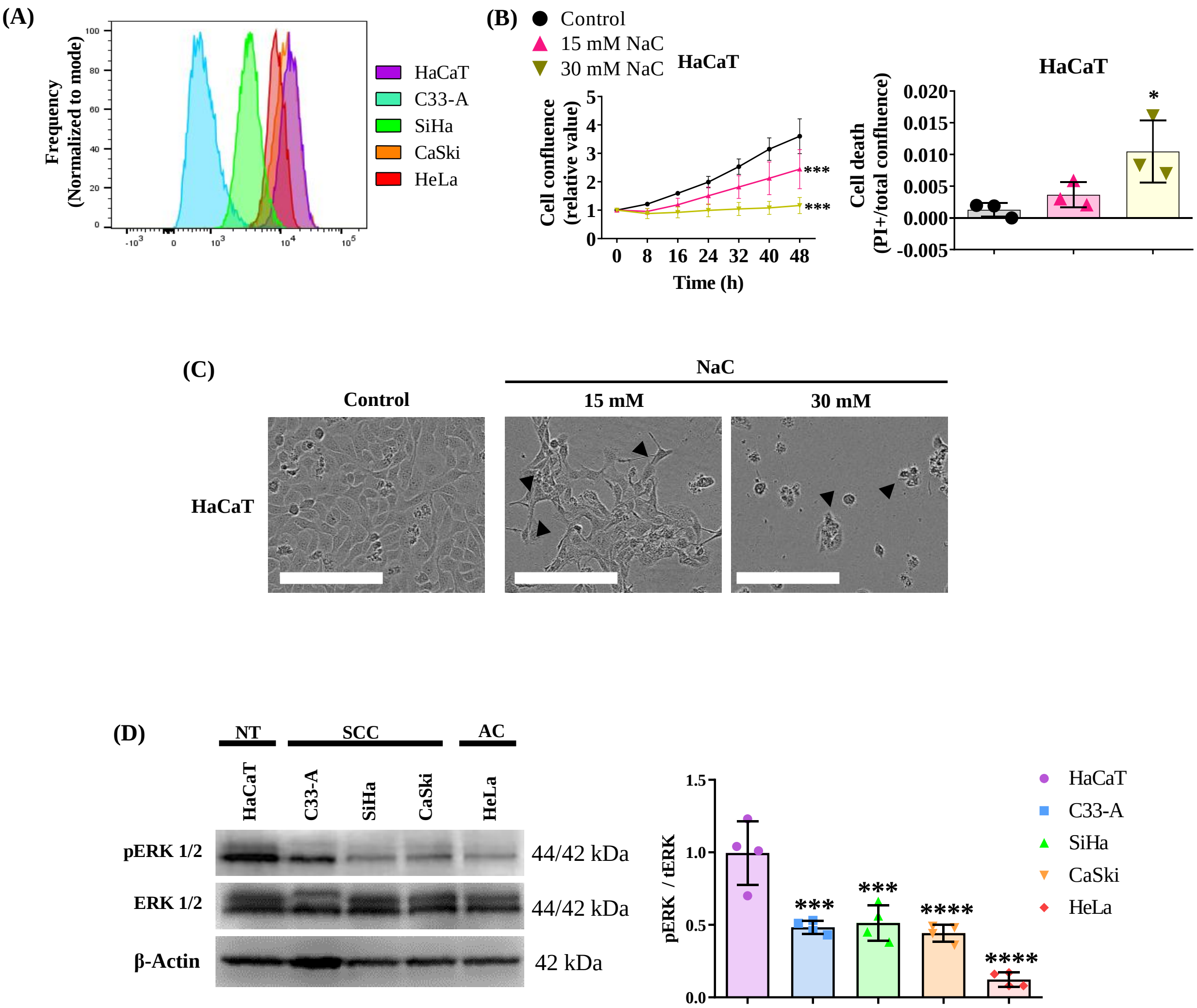

*Figure S3. HaCaT non tumorigenic cell line displays elevated ROS and ERK phosphorylation levels (A) HaCaT ROS levels were evaluated by DHE staining and flow cytometry, which revealed HaCaT is highly oxidized, similar to HeLa and CaSki cervical cancer cell lines. (B) Antioxidant treatment (NaC 15 or 30 mM) of HaCaT non tumorigenic cells caused a decrease in cell proliferation and survival at the highest concentrations used. (C) Representative photos of antioxidant treatment in HaCaT cell line following 48 hours of treatment, which suggest morphological changes indicated with black arrows. The white scale bar represents 200 µm. (D) ERK phosphorylation was also evaluated in HaCaT, which shows high ERK activation compared to CC cell lines. Graph shows mean ±SD of 3-4 experiments. One-way ANOVA or Two-way ANOVA; Tukey post-hoc. * vs Control or vs HaCaT; $p<0.05$. NT, non-tumorigenic; SCC, squamous cell carcinoma; AC, adenocarcinoma; NaC, N-acetylcysteine; pERK, phosphorylated ERK; tERK, total ERK; kDa, kilodalton.*

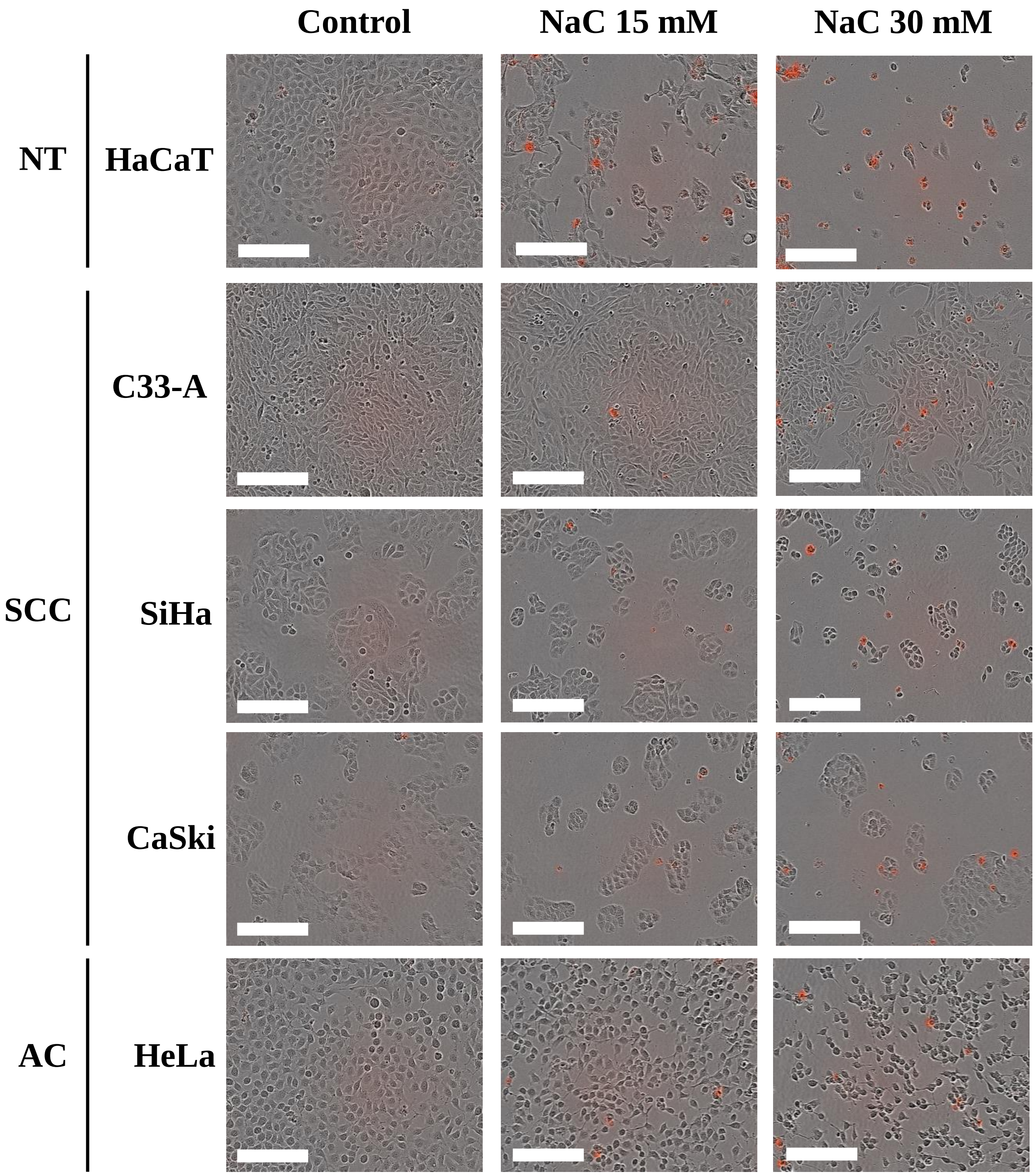

Figure S4. Antioxidant treatment induces cell death in CC cell lines and normal keratinocytes. Representative images showing cell death evaluation using propidium staining in HaCaT, C33A, SiHa, CaSki and HeLa cell lines following 48 hours of NaC treatment (15 or 30 mM) . Antioxidant treatment resulted in cell death at the highest concentration tested across all cell lines. The white scale bar represents 200 µm. NT, non-tumorigenic; SCC, squamous cell carcinoma; AC, adenocarcinoma; NaC, N-acetylcysteine

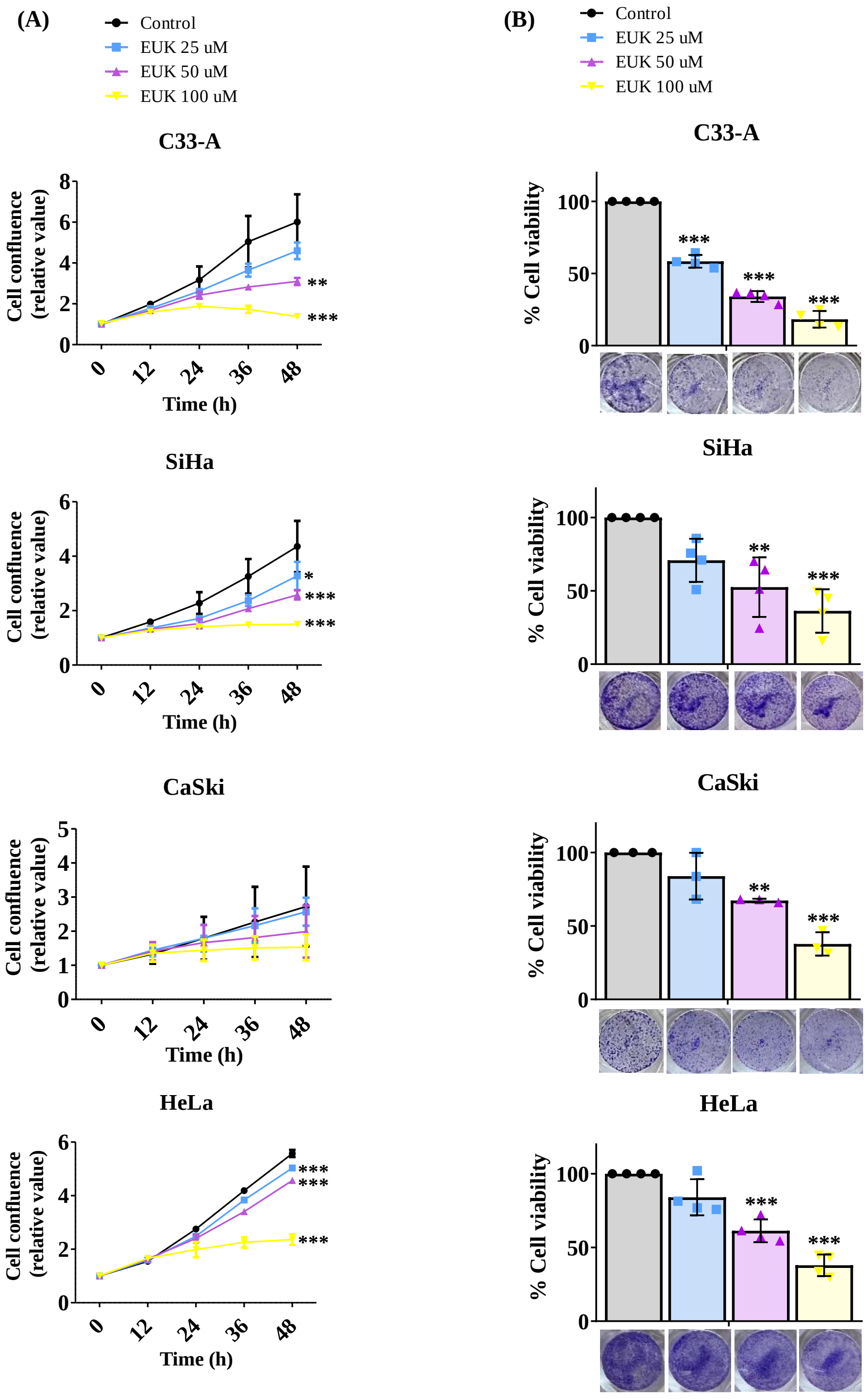

*Figure S5. EUK 134 treatment decreased proliferation and viability on CC cells. (A) CC cell lines were treated with Superoxide dismutase (SOD) mimetic, EUK 134 (25, 50 or 100 µm), for 48 hours at the specified concentrations, and proliferation was evaluated using real time imaging System. (B) To evaluate viability, cells were treated at the same concentrations of EUK 134 and after 48 hours, crystal violet assay was performed, showing decreased viability in CC cell lines. Graph shows mean ±SD of 3-4 experiments. One-way ANOVA or Two-way ANOVA; Tukey post hoc. * vs Control; p<0.05.*

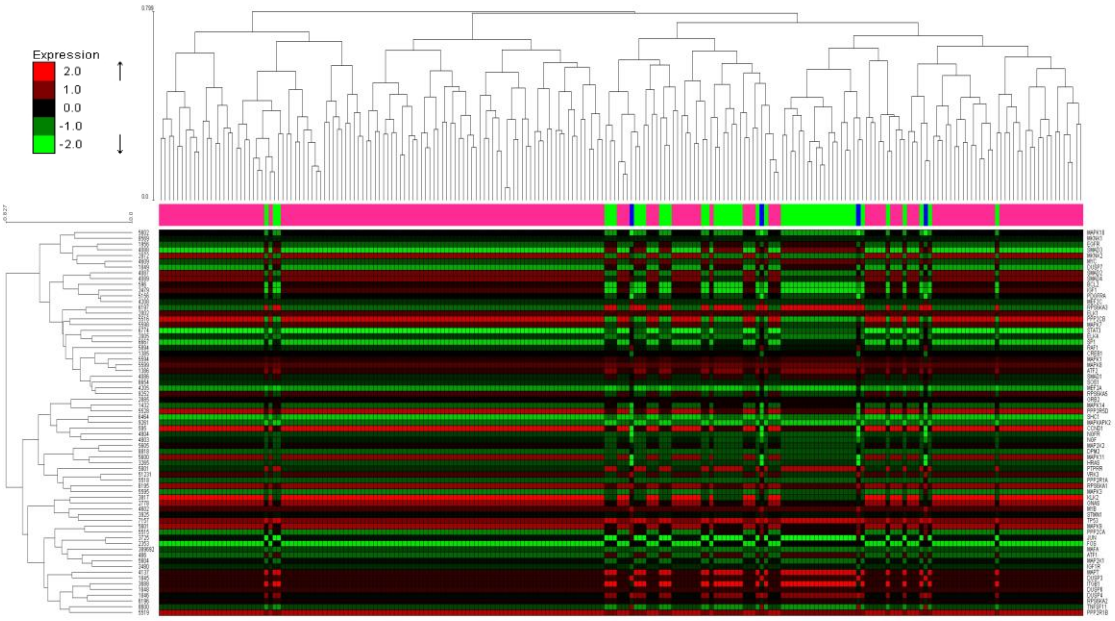

Figure S6. Unsupervised hierarchical clustering displays differences between histological CC types according to an ERK activation gene signature. ERK gene signature was analyzed in a TCGA CC sample database, and an unsupervised hierarchical clustering was performed, revealing two major clusters, one enriched in squamous cervical carcinoma (pink) and a second one enriched by cervical adenocarcinoma and adenoesquamous samples (green and blue, respectively).

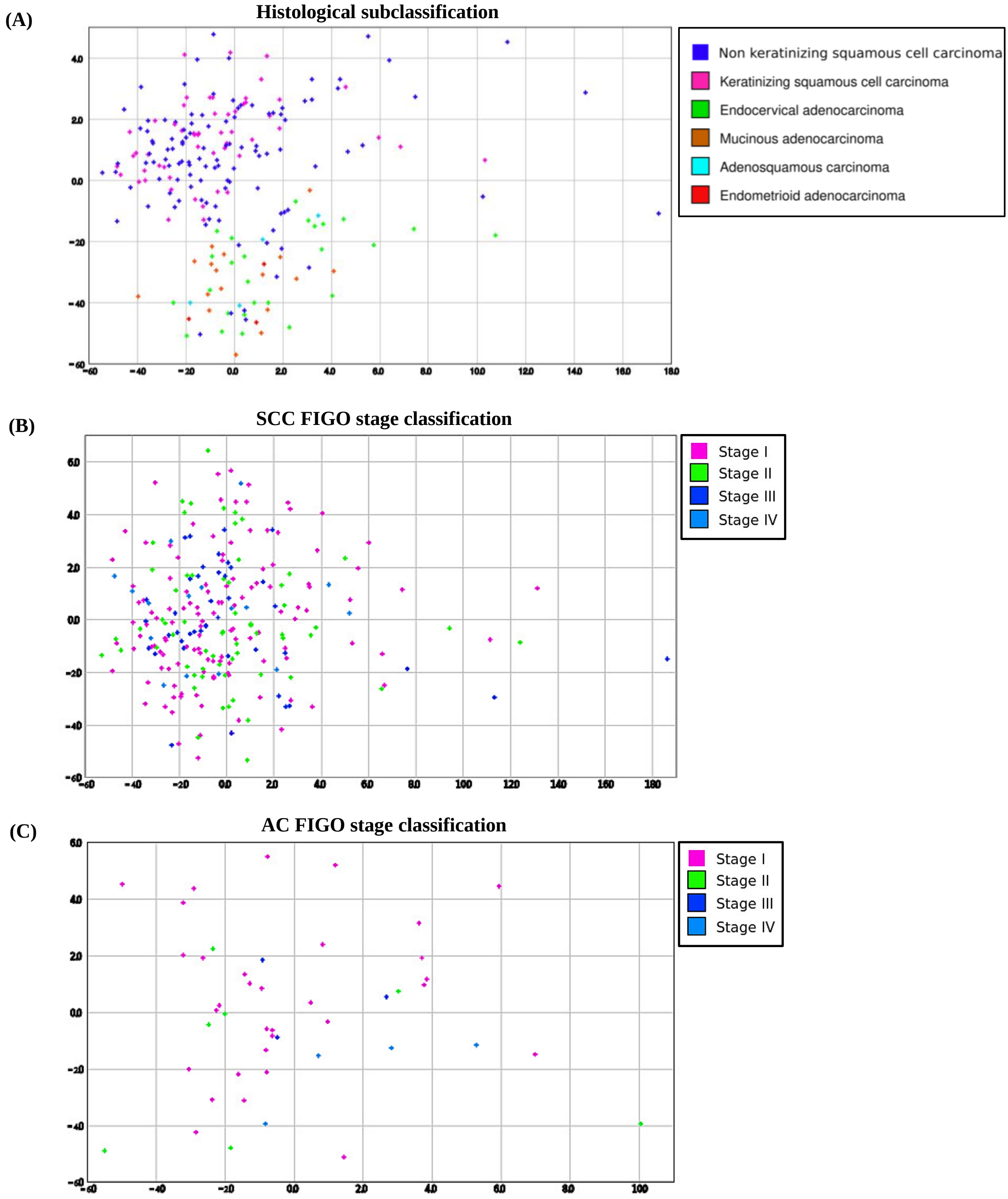

Figure S7. ERK activation gene signature does not separate CC tumor samples in function of histological subclassification or stage of the disease. (A) The ERK activation gene signature was analyzed in CC tumor samples categorized by histological subclassification, but no groups were observed according to this classification. (B, C) Samples were also separated by histological subtype and classified by FIGO stage, the ERK activation related signature was analyzed and PCA was performed, but no clusters were observed. SCC, squamous cell carcinoma; AC, adenocarcinoma.

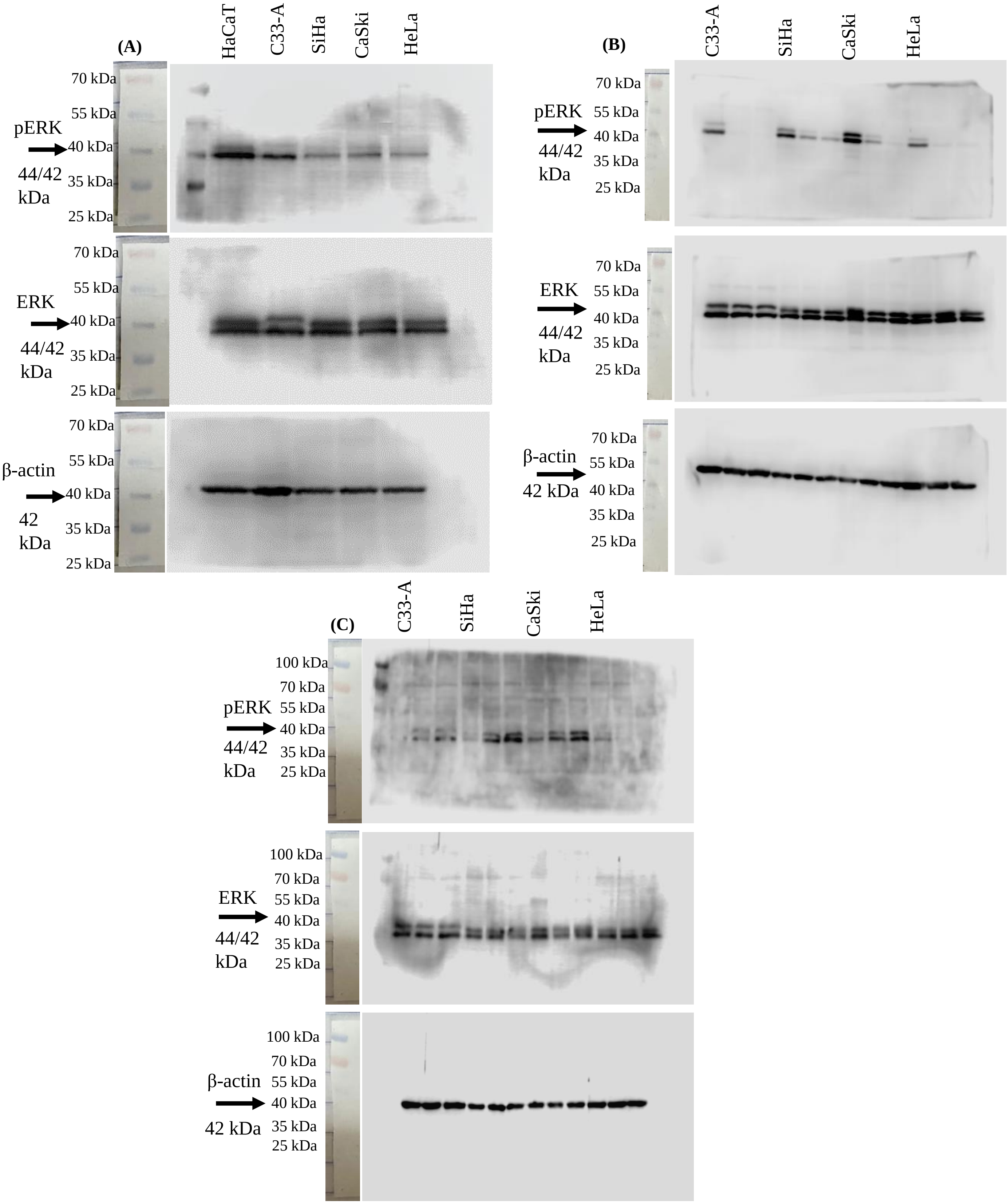

Figure S8. Complete blots for pERK, tERK and β-actin. (A) Blots showing basal levels of pERK, ERK and β-actin in CC cell lines from Fig.4B (HaCaT is not included in the original figure) and S3D (which includes HaCaT). (B) Blots displaying pERK, ERK and β-actin levels following 1 hour treatment with MEKi (25 or 50 nM) as shown in Fig. 4C. (C) Blots showing pERK, ERK and β-actin levels following 24 hours of NaC treatment (15 or 30 mM) as shown in Fig. 5A. pERK, tERK and β-actin were revealed on the same membrane. pERK, phosphorylated ERK; tERK, total ERK; kDa, kilodalton.

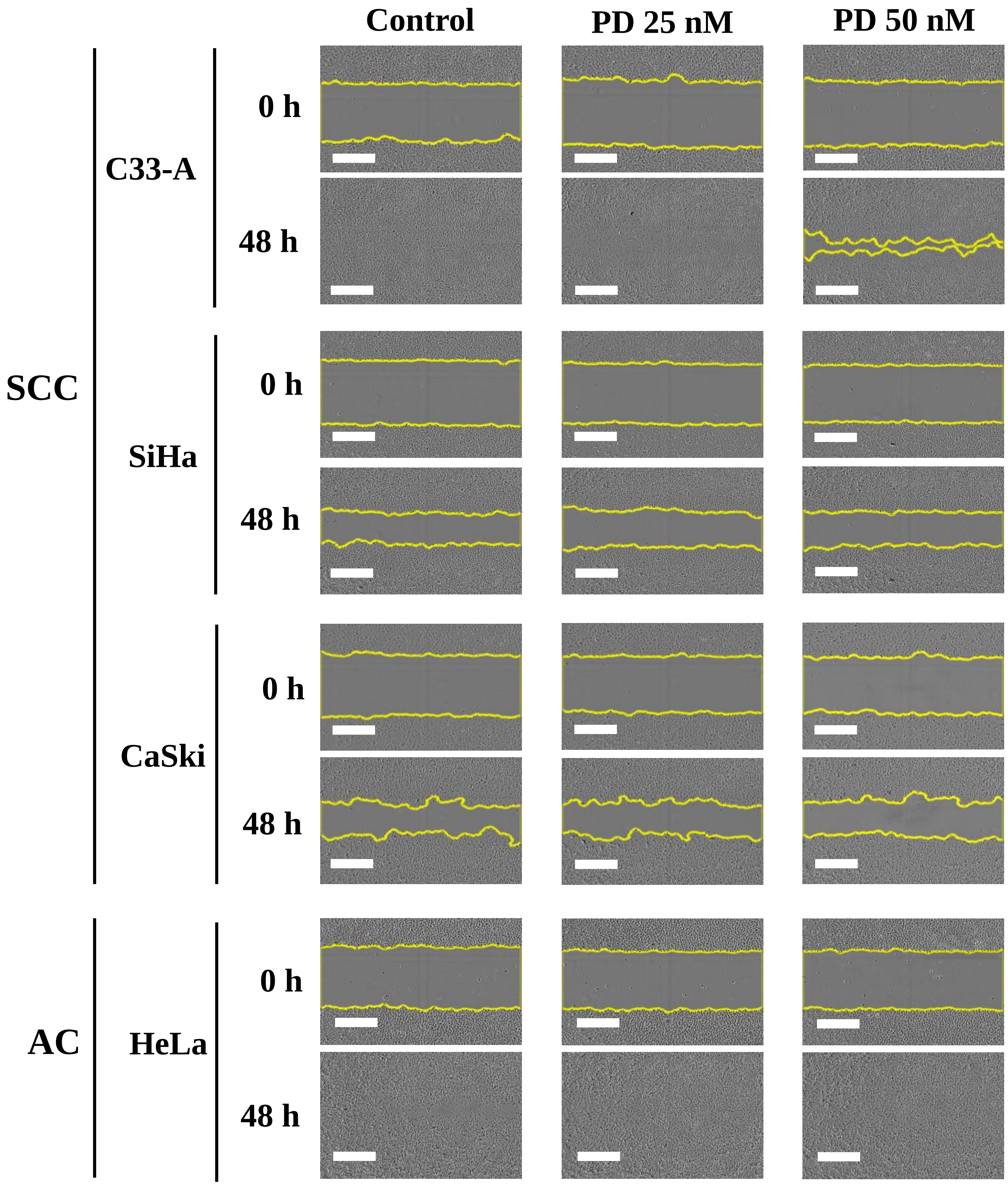

Figure S9. MEKi treatment decreased cell migration on SCC CC cell lines. Representative images taken using a real-time imaging System at 0 or 48 hours post treatment with MEKi 25 or 50 nM are shown. The yellow line represent the border of the wound set by the Incucyte 96-well cell migration/ invasion software. The white scale bar represents 400 µm. SCC, squamous cell carcinoma; AC, adenocarcinoma.

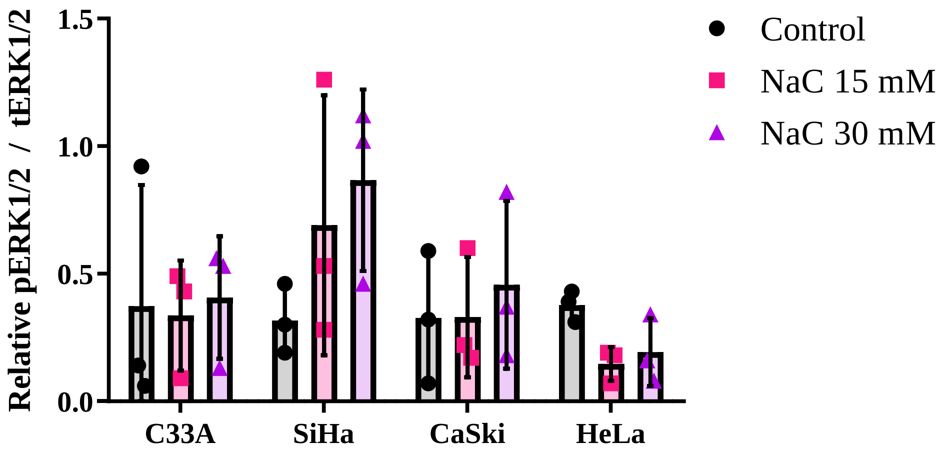

*Figure S10. pERK levels following N-acetylcysteine treatment in CC cells. pERK levels were evaluated through western blot after 24 hours of treatment with NaC (15 or 30 mM) across all CC cell lines. The quantification depicted in the graph shows an increasing trend in pERK following NaC treatment in the SiHa cell line, whereas a decreasing trend is observed in the AC cell line HeLa. No statistical differences were observed. Graph shows mean ±SD of 3 independent experiments. One-way ANOVA; Tukey post-hoc. * vs Control; $p<0.05$.*
